# Supplementary material for: Catalytic Acceptorless Dehydrogenation (CAD) of Secondary Benzylic Alcohols into Value-Added Ketones Using Pd(II)–NHC Complexes
Source: Molecules. 2023 Jun 25;28(13):4992. doi: 10.3390/molecules28134992 (PMC10343575; doi:10.3390/molecules28134992)
Supplement: Supplementary file 1 [file molecules-28-04992-s001.zip › molecules-2433079-supplementary.pdf]

Electronic Supplementary Information

Catalytic Acceptorless Dehydrogenation (CAD) of Secondary Benzylic Alcohols into Value-added Ketones using Pd(II)-NHC Complexes

Abeer Nasser Al-Romaizan [1,†], Manoj Kumar Gangwar [2,†], Ankit Verma [2,†], Salem M. Bawaked [1], Tamer S. Saleh [3,*], Rahmah H. Al-Ammari [1], Ray J. Butcher [4], Ibadur Rahman Siddiqui [2] and Mohamed Mokhtar M. Mostafa [1,*]

[1] Department of Chemistry, Faculty of Science, King Abdul-Aziz University, P.O. Box 80203, Jeddah 21589, Saudi Arabia
[2] Department of Chemistry, Faculty of Science, University of Allahabad (AoU), Prayagraj 211002, Uttar Pradesh, India
[3] Department of Chemistry, College of Science, University of Jeddah, P.O. Box 80327, Jeddah 21959, Saudi Arabia
[4] Department of Chemistry, Howard University, Washington, DC 20059, USA
* Correspondence: tamsaid@yahoo.com (T.S.S.); mmoustafa@kau.edu.sa (M.M.M.M.)
† These authors contributed equally to this work.

NMR Spectra of all the acceptorless dehydrogenation products.

General considerations:

Unless otherwise noted, all commercially available substances were used exactly as they were given. Using $CDCl_3$ solvent, ^{1}H NMR and $^{13}C\{^{1}H\}$ NMR measurements were taken on Bruker 400 MHz and 500 MHz spectrometers. Relative to TMS, chemical shifts (δ) are given in ppm, and coupling constants (J) are given in Hz. The chemical shifts and solvent signals that were used as references were converted to the TMS scale ($CDCl_3$, δC 77.0 ppm, δH 7.26 ppm). Using commercial aluminium sheets precoated with silica gel, analytical thin layer chromatography (TLC) was used to track all of the reactions. Silica gel (Merck, 200−400 mesh) was used for column chromatography. Singlet singlet (s), doublet (d), triplet (t), quartet (q), doublet of doublet (dd), doublet of triplet (dt), triplet of triplet (tt), multiplet (m) etc, are the abbreviations used for ^{1}H NMR spectra to denote the signal multiplicity. Using an Agilent Technologies 7980A GC system with an HP-5 column and FID detector, GC analysis was done to confirm H2 evolution in the reaction. The YL 6100GC, which has a thermal conductivity detector and a Sigma-Aldrich Metal Packed GC Column, can detect evolved H2 (13407-U SUPELCO).

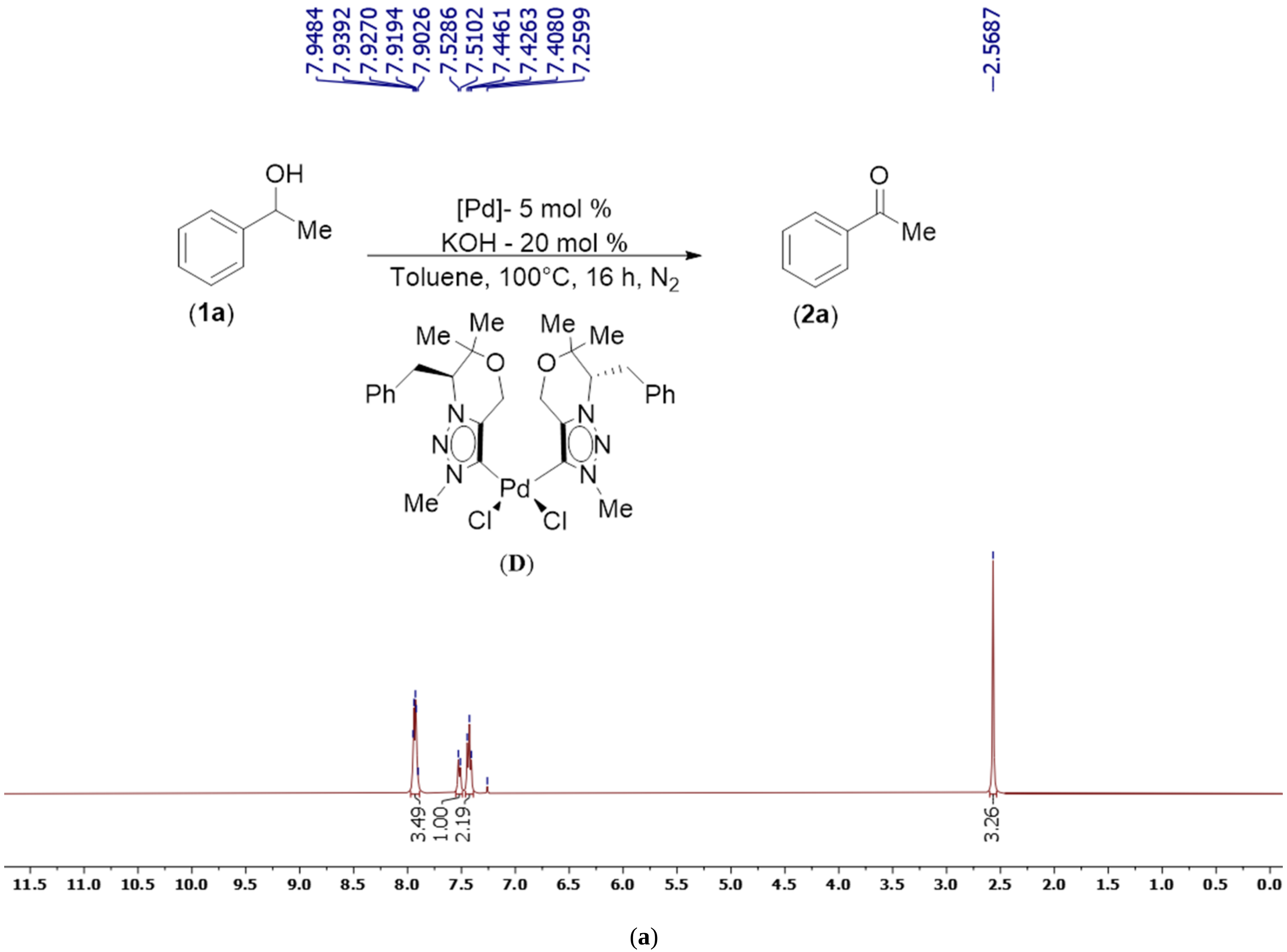
7.9484
7.9392
7.9270
7.9194
7.9026
7.5286
7.5102
7.4461
7.4263
7.4080
7.2599
2.5687
OH
Me
[Pd]- 5 mol %
KOH - 20 mol %
Toluene, 100°C, 16 h, N2
O
Me
(1a)
(2a)
Me
Me
Me
Me
O
O
Ph
Ph
N
N
N
N
N
N
Pd
Me
Me
Cl
Cl
(D)
3.49
1.00
2.19
3.26
11.5
11.0
10.5
10.0
9.5
9.0
8.5
8.0
7.5
7.0
6.5
6.0
5.5
5.0
4.5
4.0
3.5
3.0
2.5
2.0
1.5
1.0
0.5
0.0

(**a**)

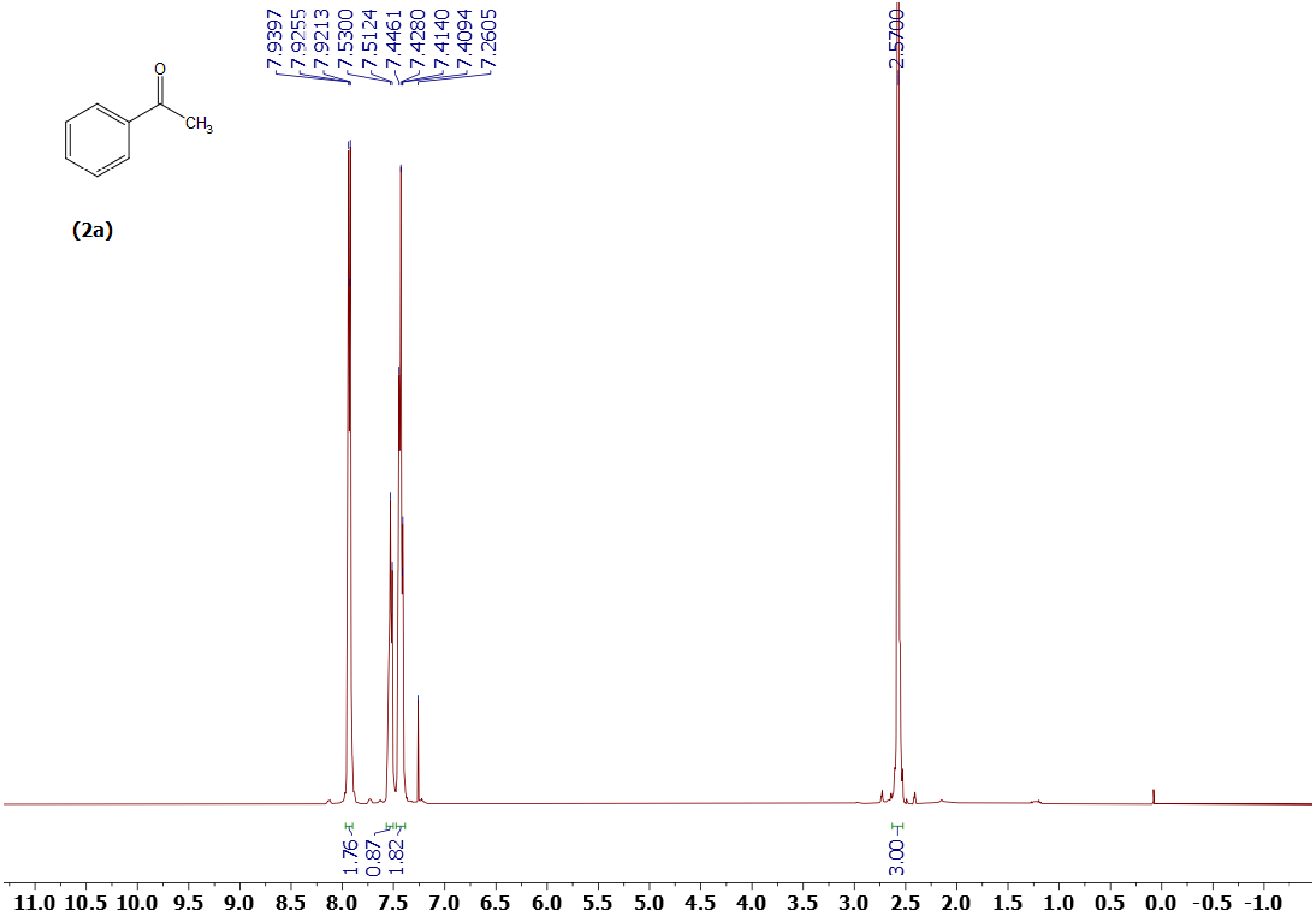

O
CH3
(2a)
7.9397
7.9255
7.9213
7.5300
7.5124
7.4461
7.4280
7.4140
7.4094
7.2605
2.5700
1.76
0.87
1.82
3.00
11.0 10.5 10.0 9.5 9.0 8.5 8.0 7.5 7.0 6.5 6.0 5.5 5.0 4.5 4.0 3.5 3.0 2.5 2.0 1.5 1.0 0.5 0.0 -0.5 -1.0

(**b**)

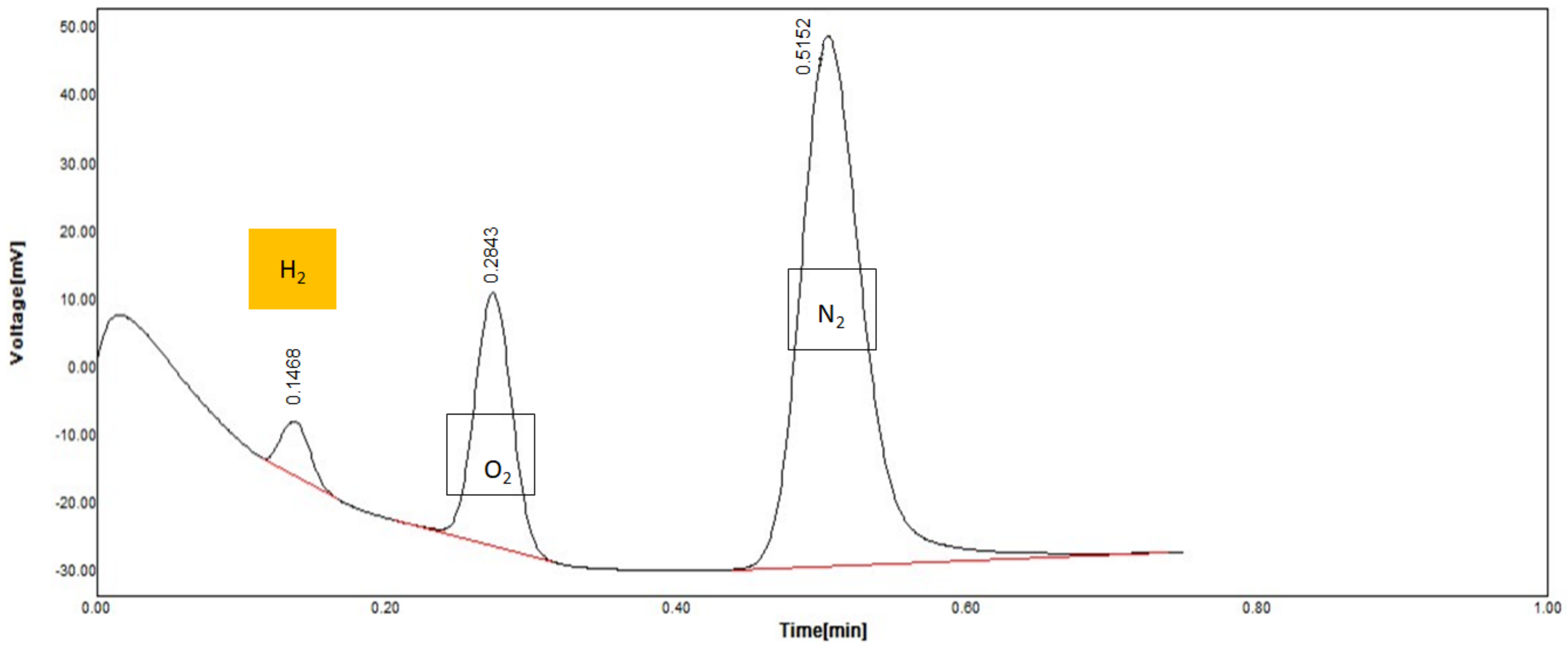

(c)

Figure S1. (**a**) ^{1}H NMR spectrum of **2a** in $CDCl_3$. (**b**) Crude ^{1}H NMR spectrum of **2a** in $CDCl_3$. (**c**) GC traces after 24 hours reaction to confirm H_2 evolution.

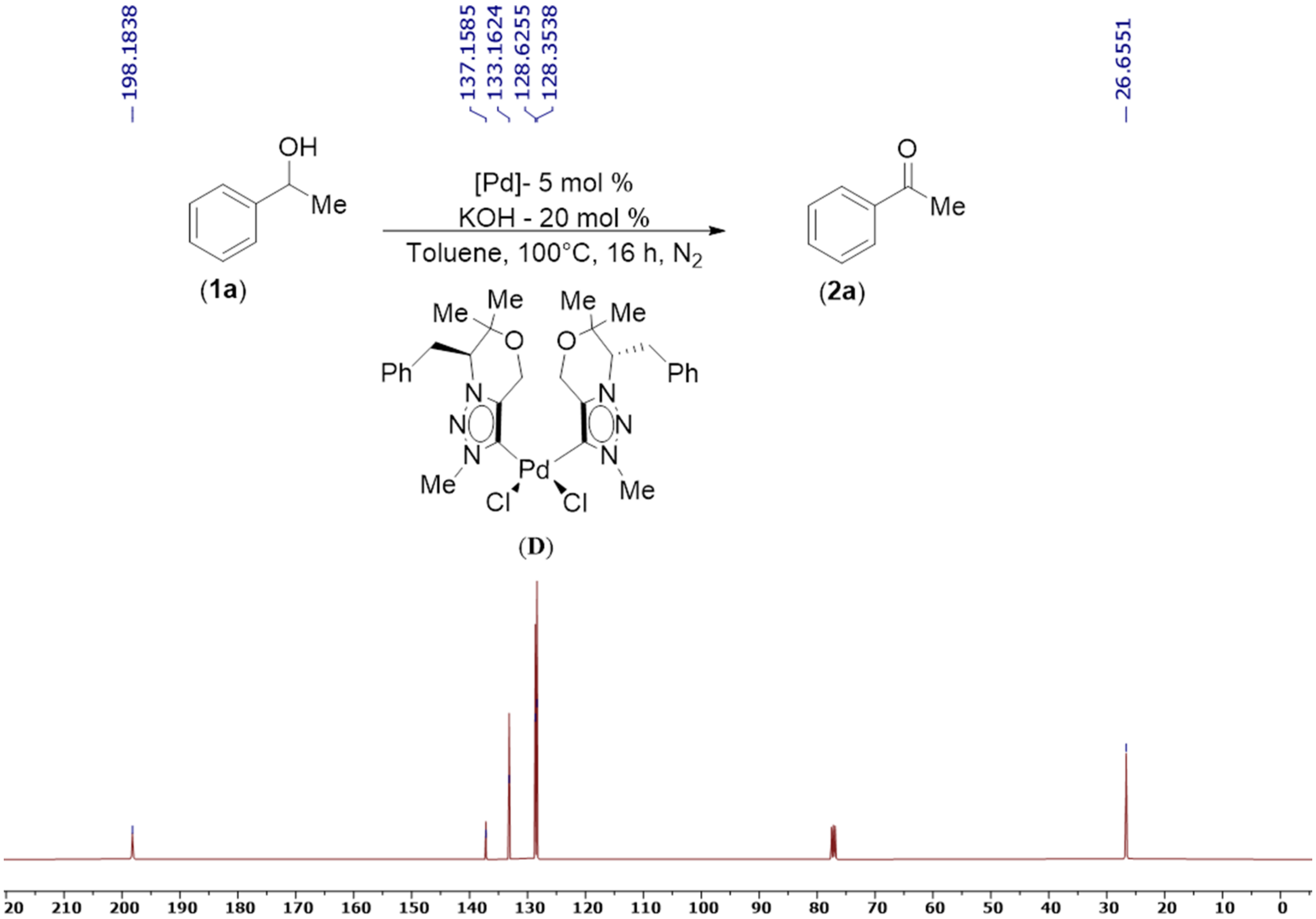

Figure S2. $^{13}C\{^{1}H\}$ NMR spectrum of **2a** in $CDCl_3$.

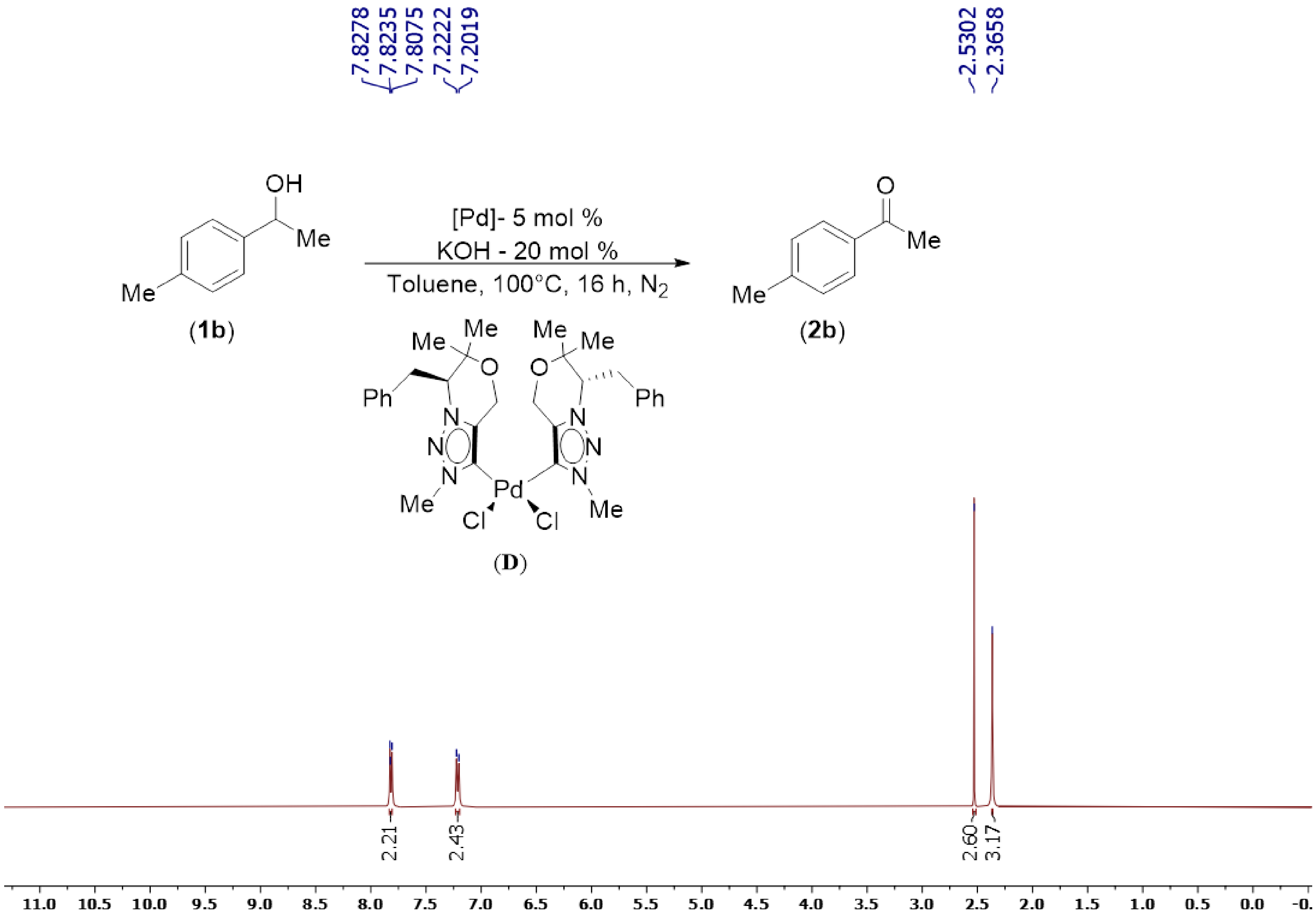

Figure S3. ^{1}H NMR spectrum of **2b** in $CDCl_3$.

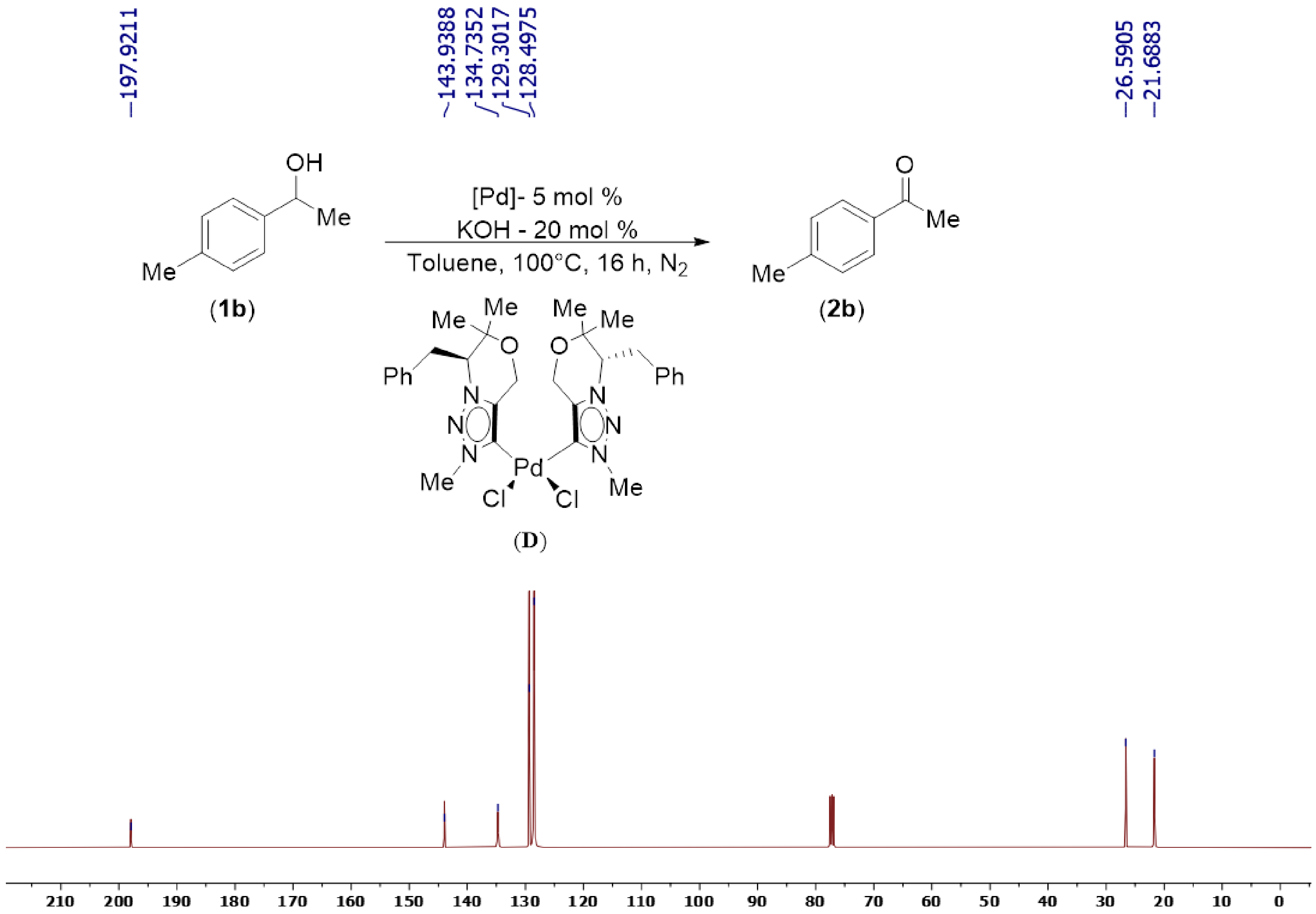

Figure S4. $^{13}C\{^{1}H\}$ NMR spectrum of **2b** in $CDCl_3$.

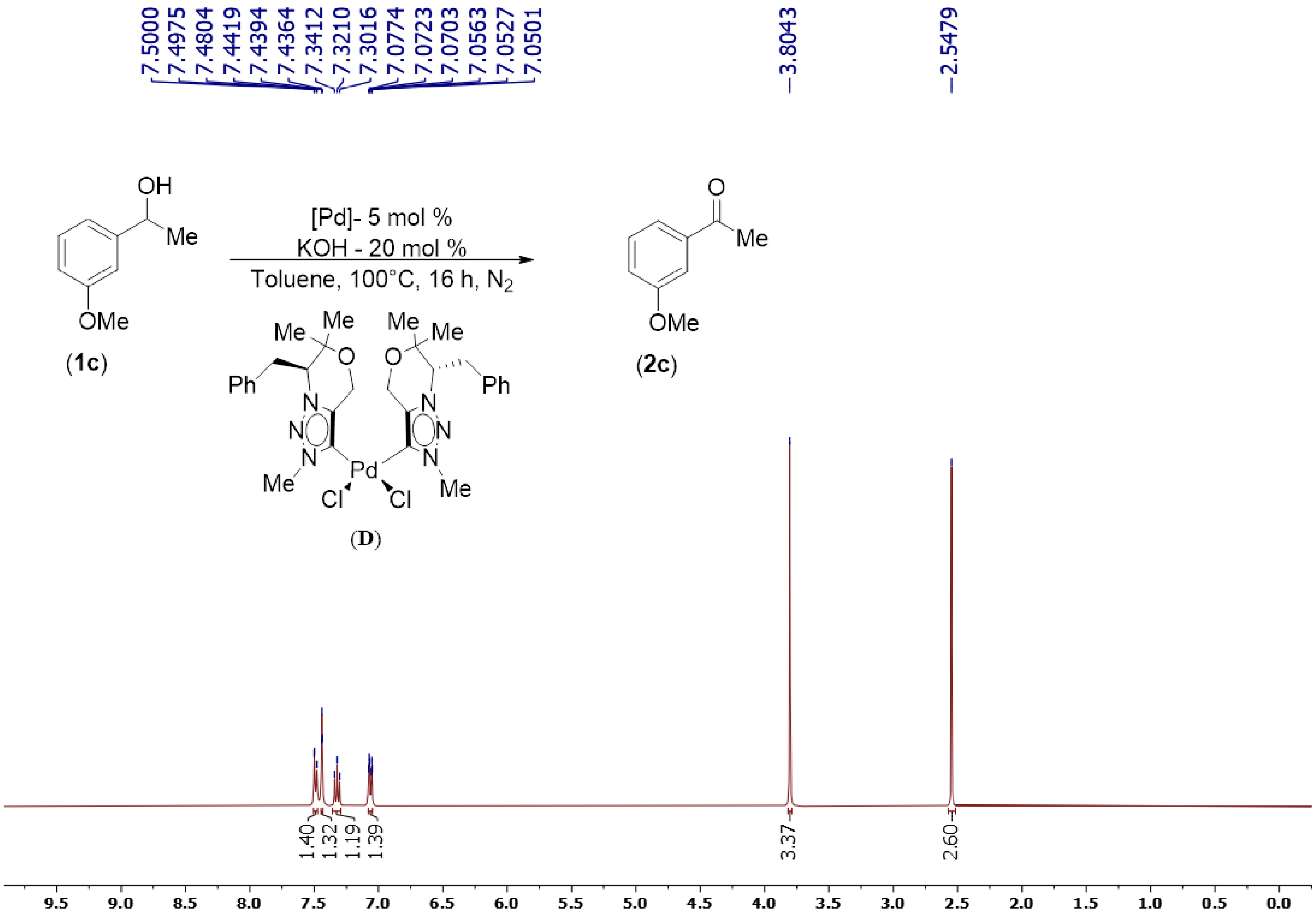

Figure S5. ^{1}H NMR spectrum of **2c** in $CDCl_3$.

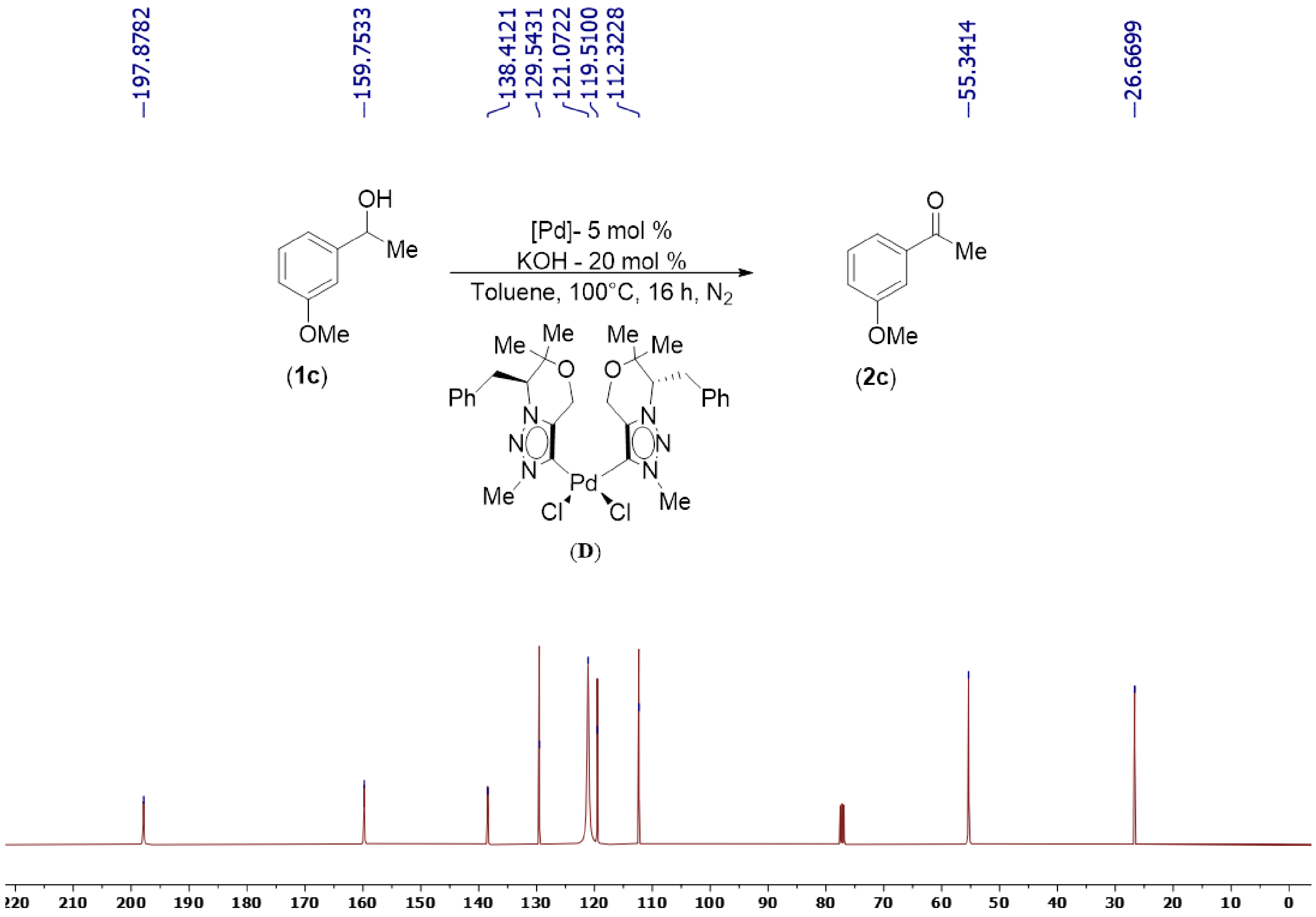

Figure S6. $^{13}C\{^{1}H\}$ NMR spectrum of **2c** in $CDCl_3$.

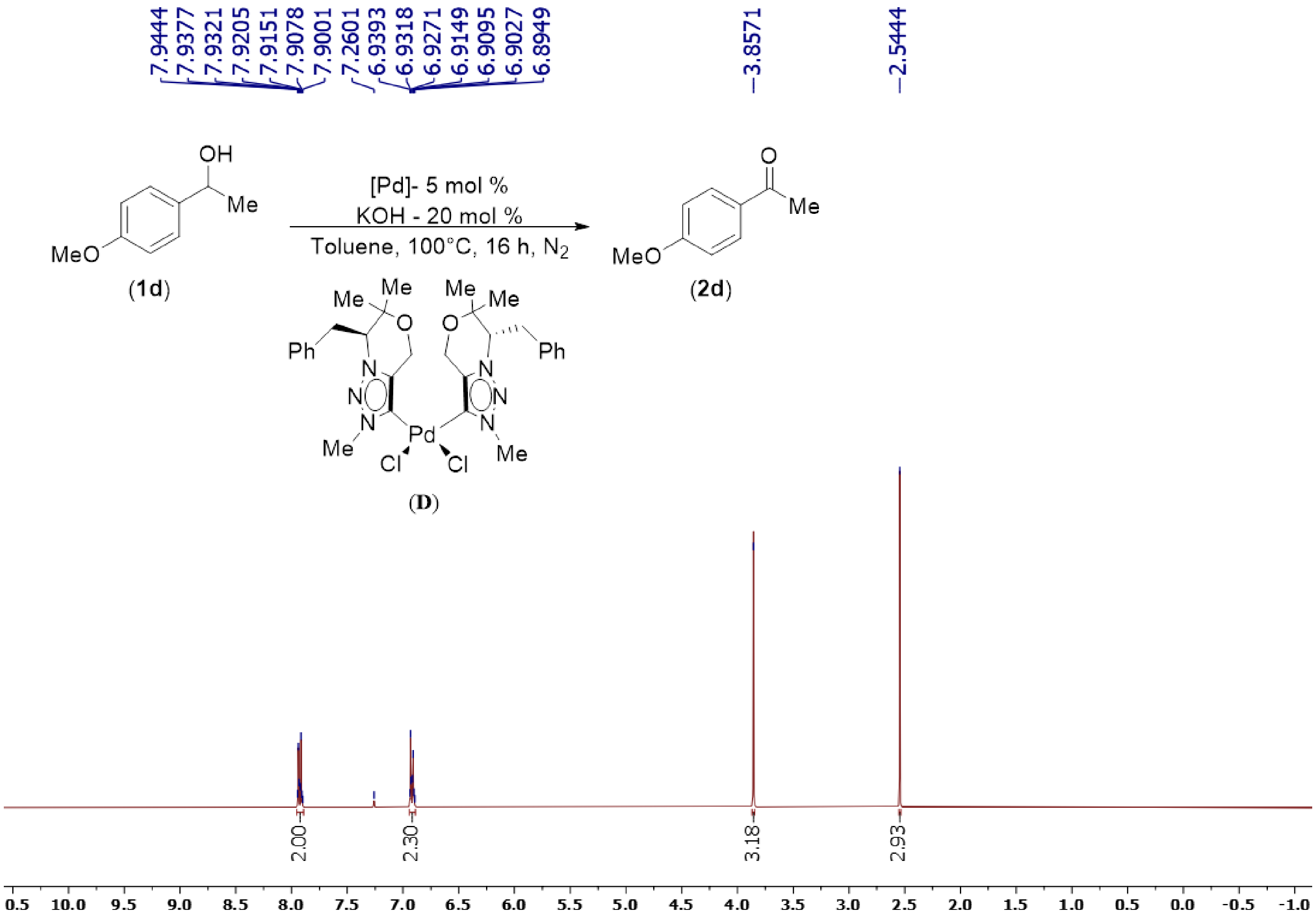

Figure S7. ^{1}H NMR spectrum of **2d** in $CDCl_3$.

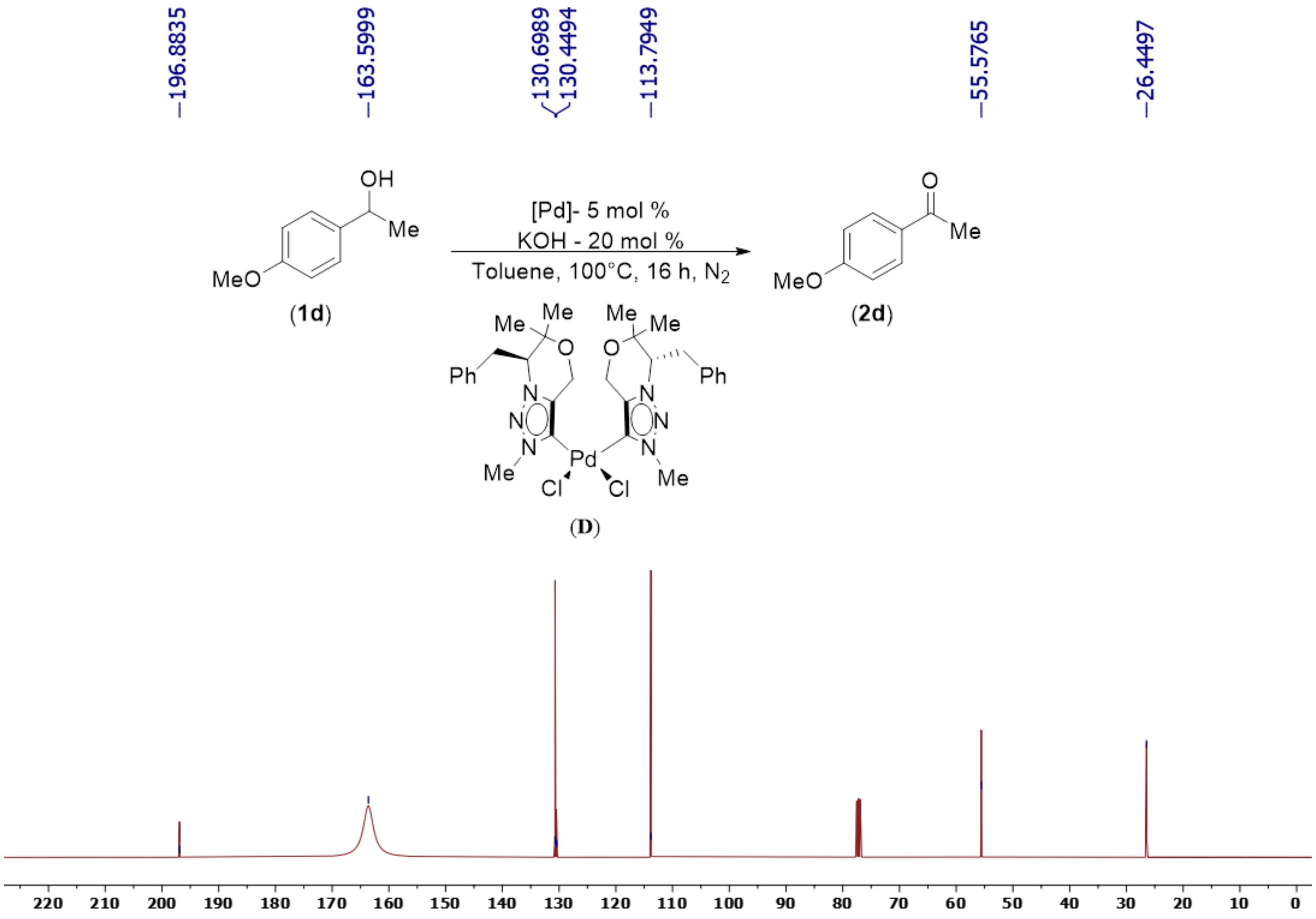

Figure S8. $^{13}C\{^{1}H\}$ NMR spectrum of **2d** in $CDCl_3$.

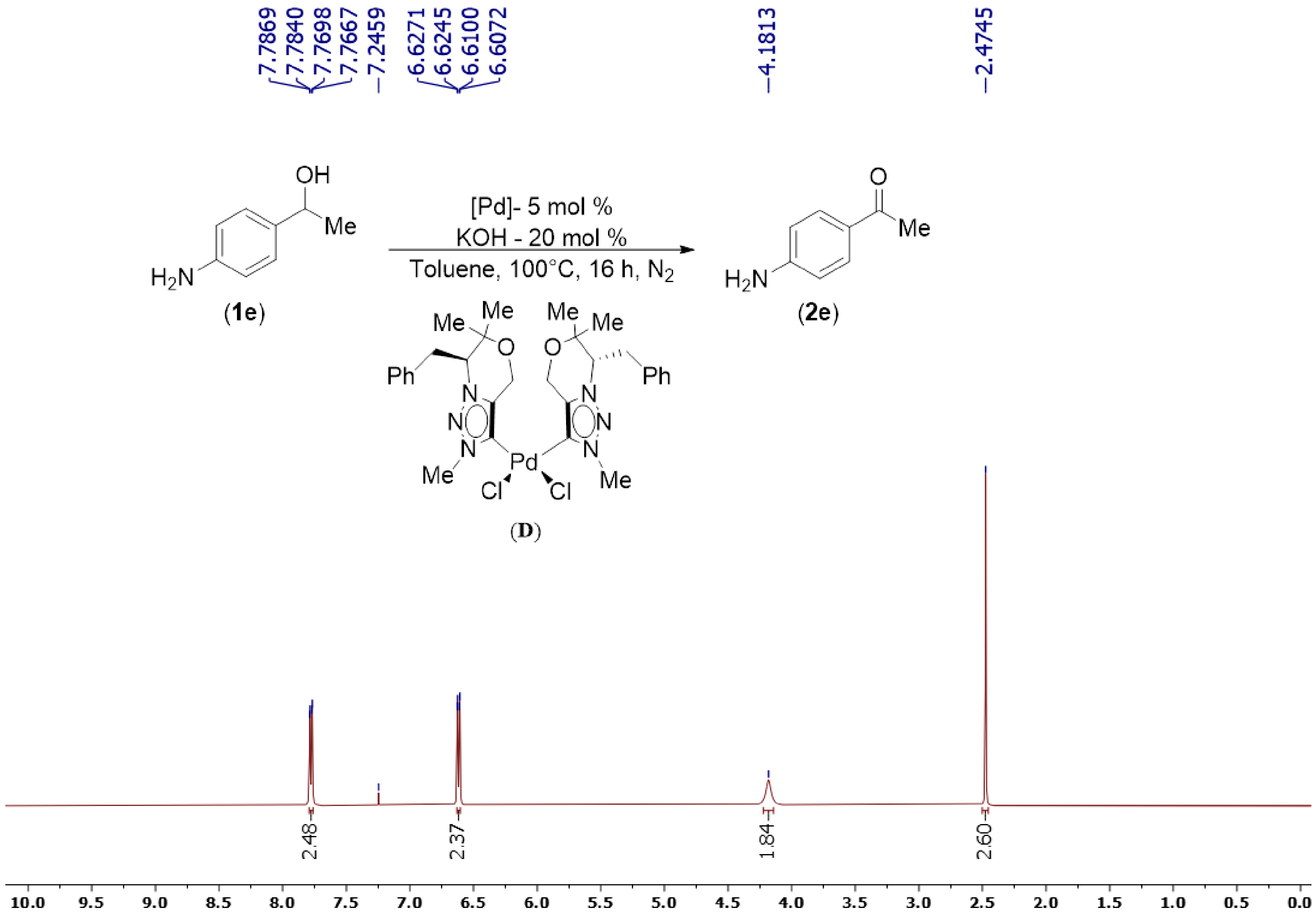

Figure S9. ^{1}H NMR spectrum of **2e** in $CDCl_3$.

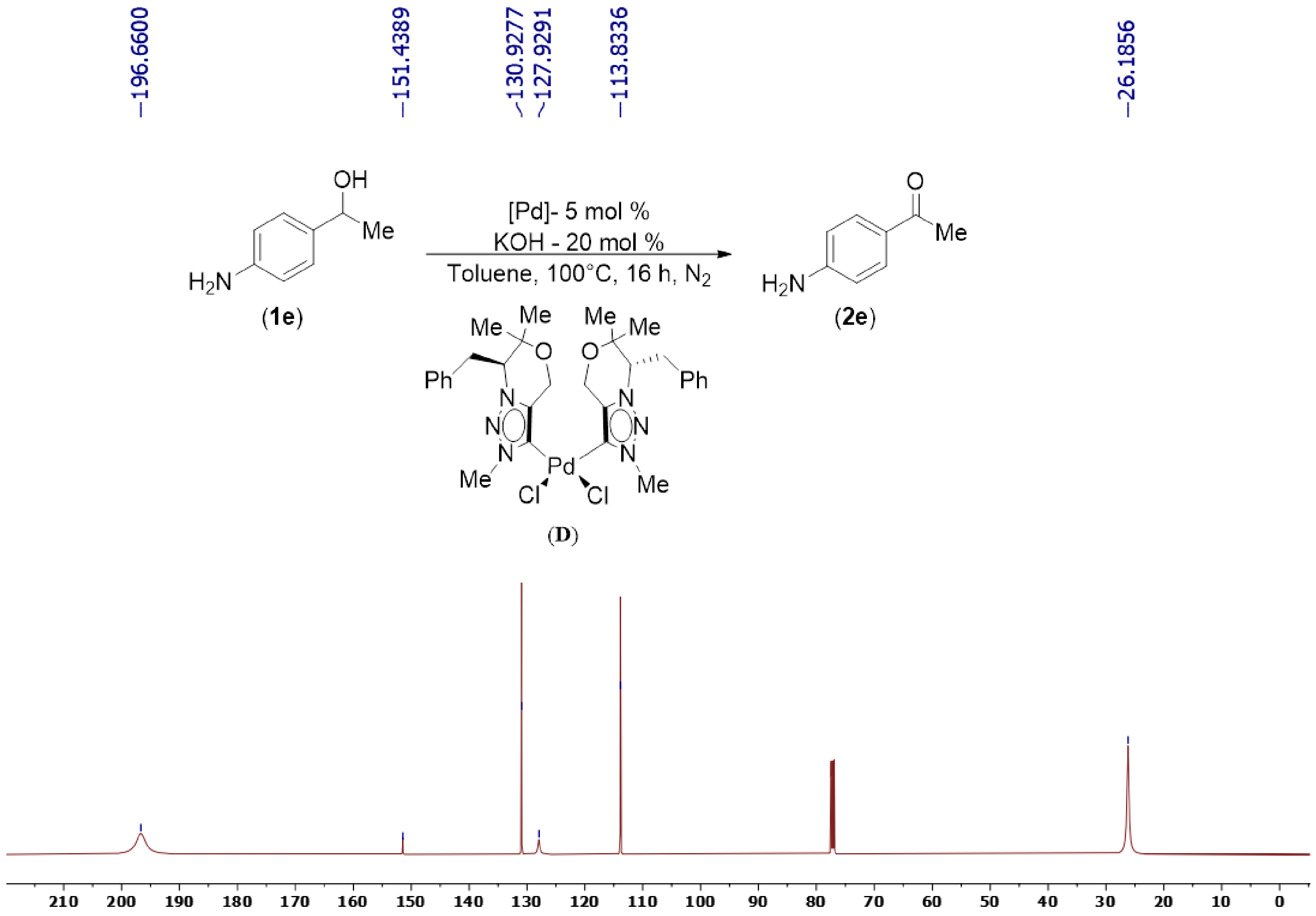

Figure S10. $^{13}C\{^1H\}$ NMR spectrum of **2e** in $CDCl_3$.

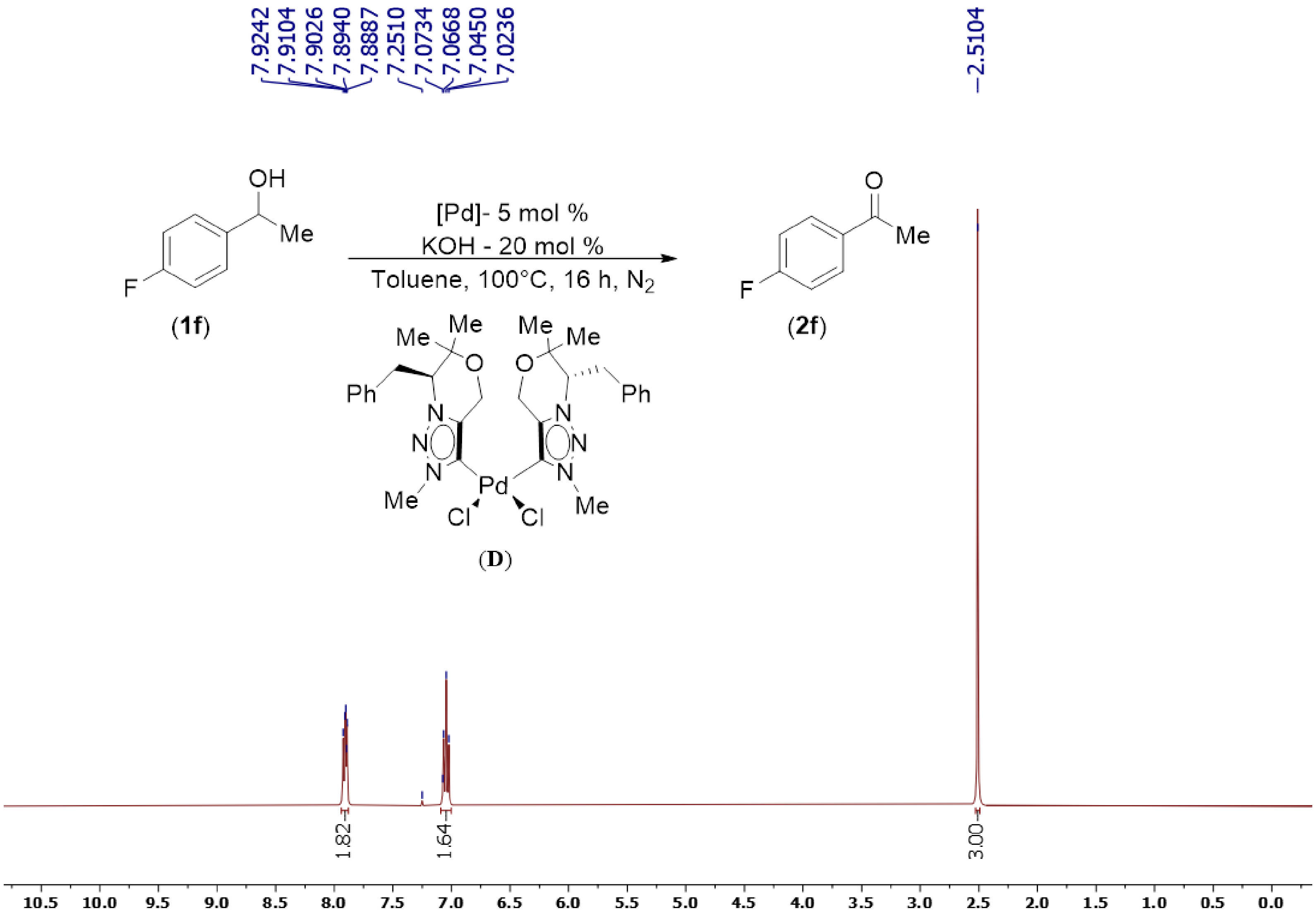

Figure S11. ^{1}H NMR spectrum of **2f** in $CDCl_3$.

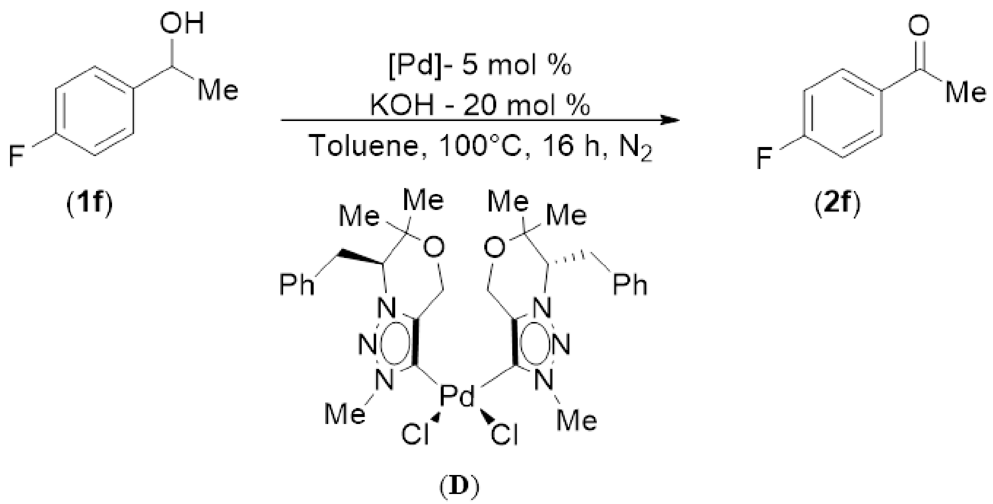

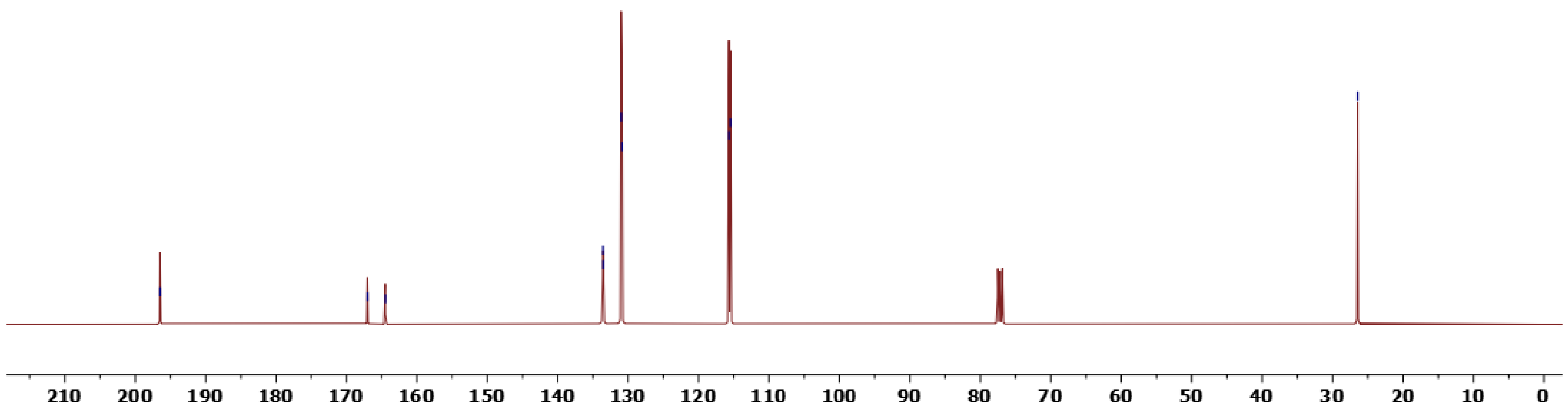

Figure S12. $^{13}C\{^1H\}$ NMR spectrum of **2f** in $CDCl_3$.

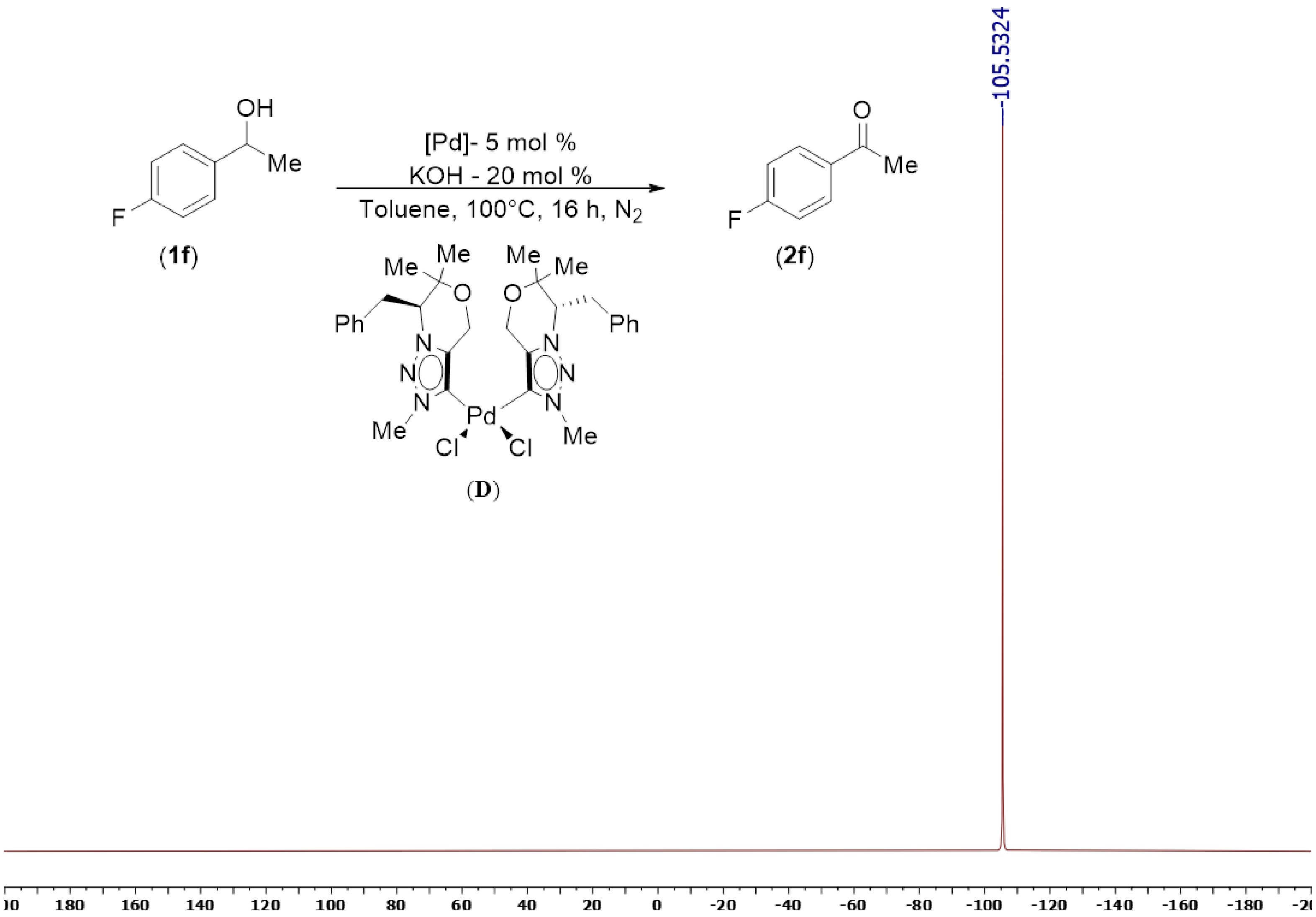

Figure S13. ^{19}F NMR spectrum of **2f** in $CDCl_3$.

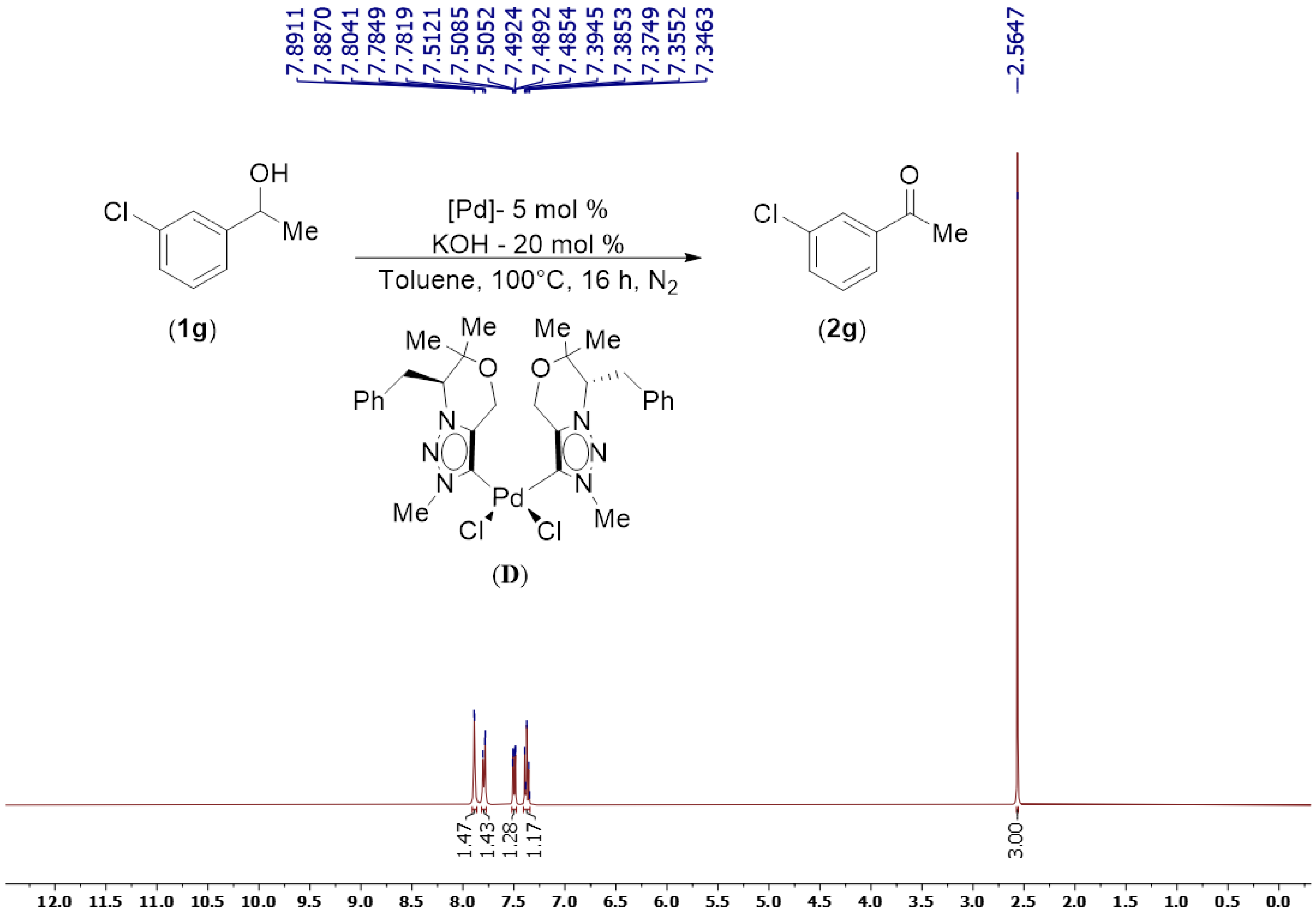

Figure S14. ^{1}H NMR spectrum of **2g** in $CDCl_3$.

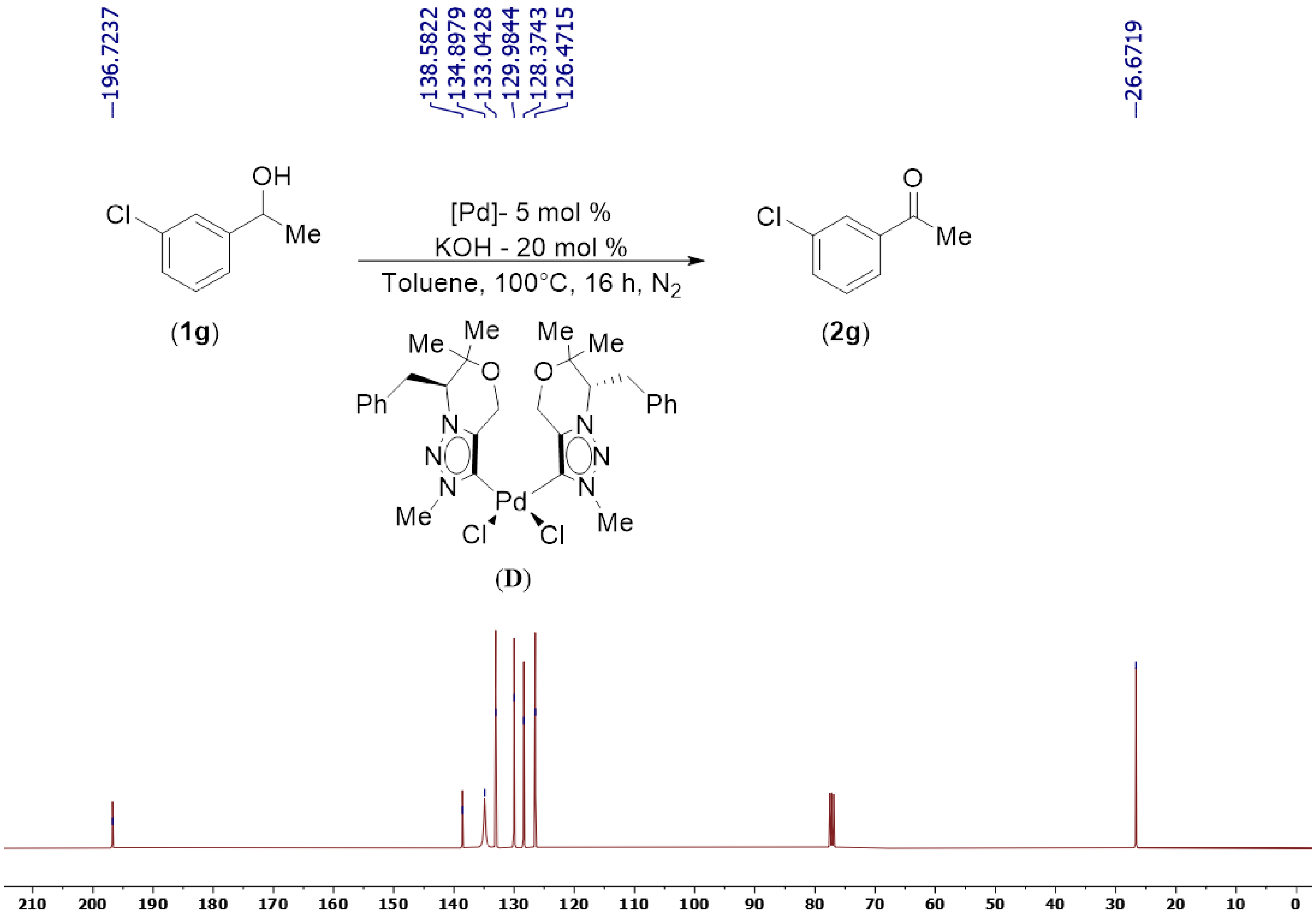

Figure S15. $^{13}C\{^1H\}$ NMR spectrum of **2g** in $CDCl_3$.

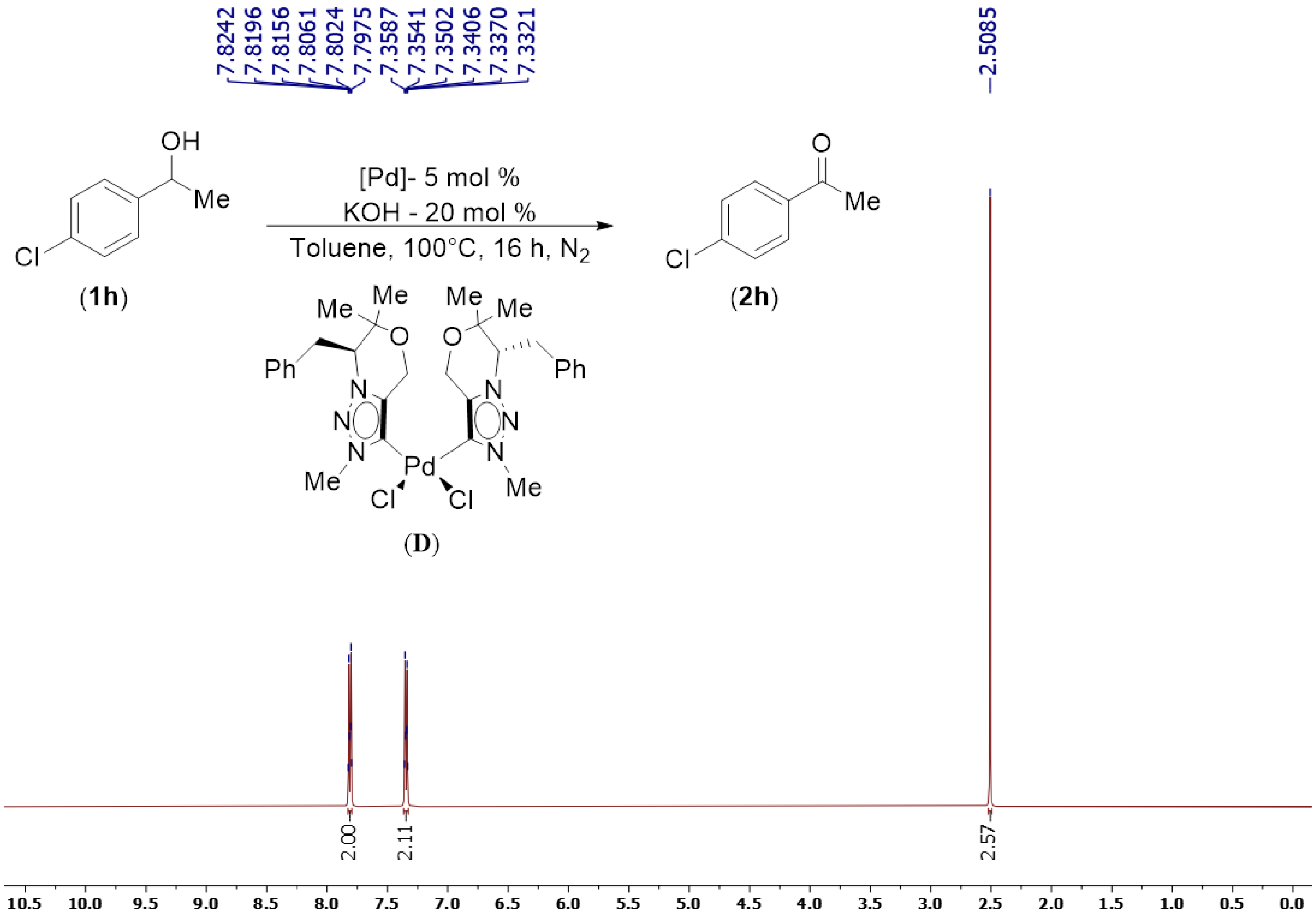

Figure S16. ^{1}H NMR spectrum of **2h** in $CDCl_3$.

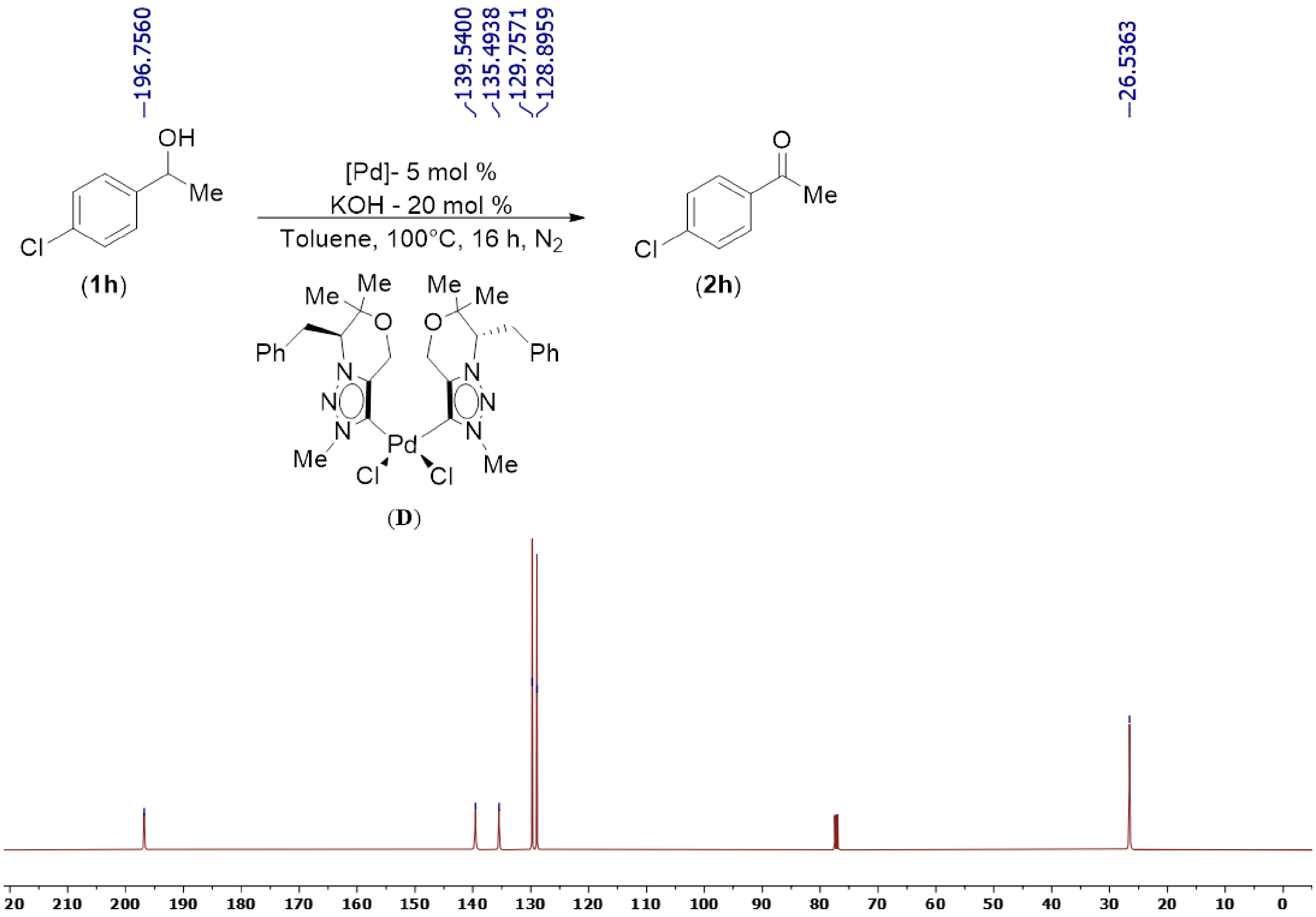

Figure S17. $^{13}C\{^{1}H\}$ NMR spectrum of **2h** in $CDCl_3$.

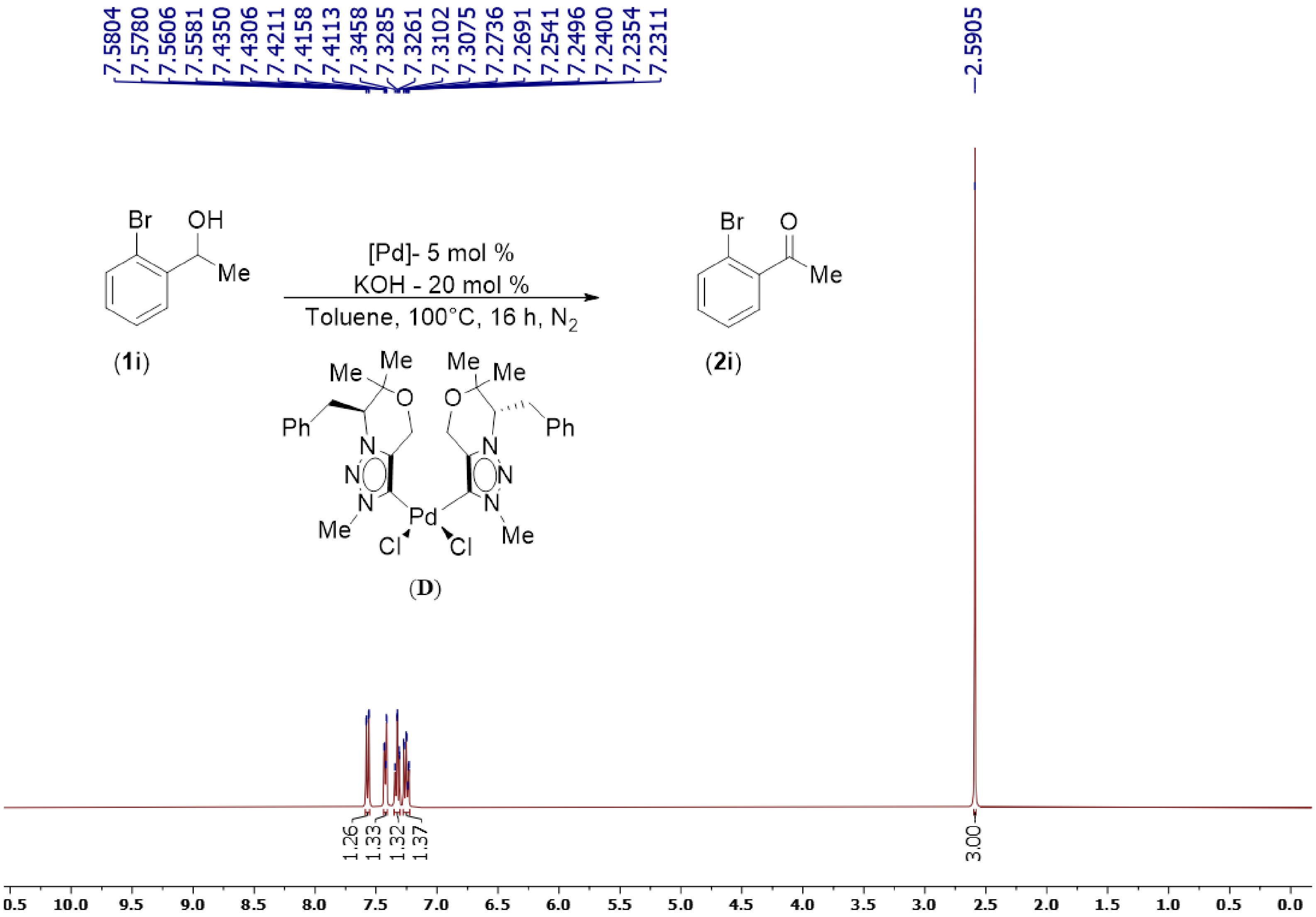

Figure S18. ^{1}H NMR spectrum of **2i** in $CDCl_3$.

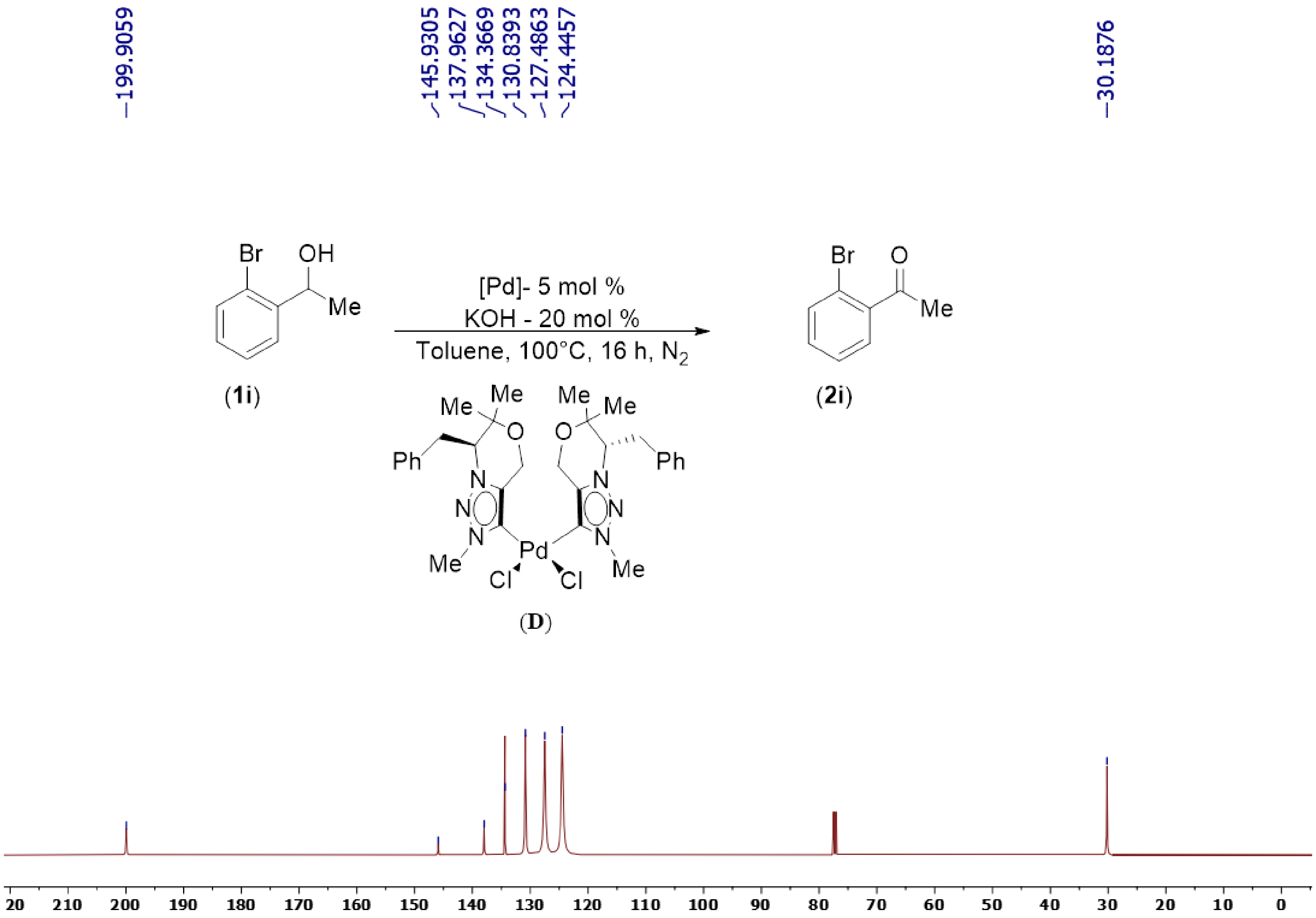

Figure S19. $^{13}C\{^{1}H\}$ NMR spectrum of **2i** in $CDCl_3$.

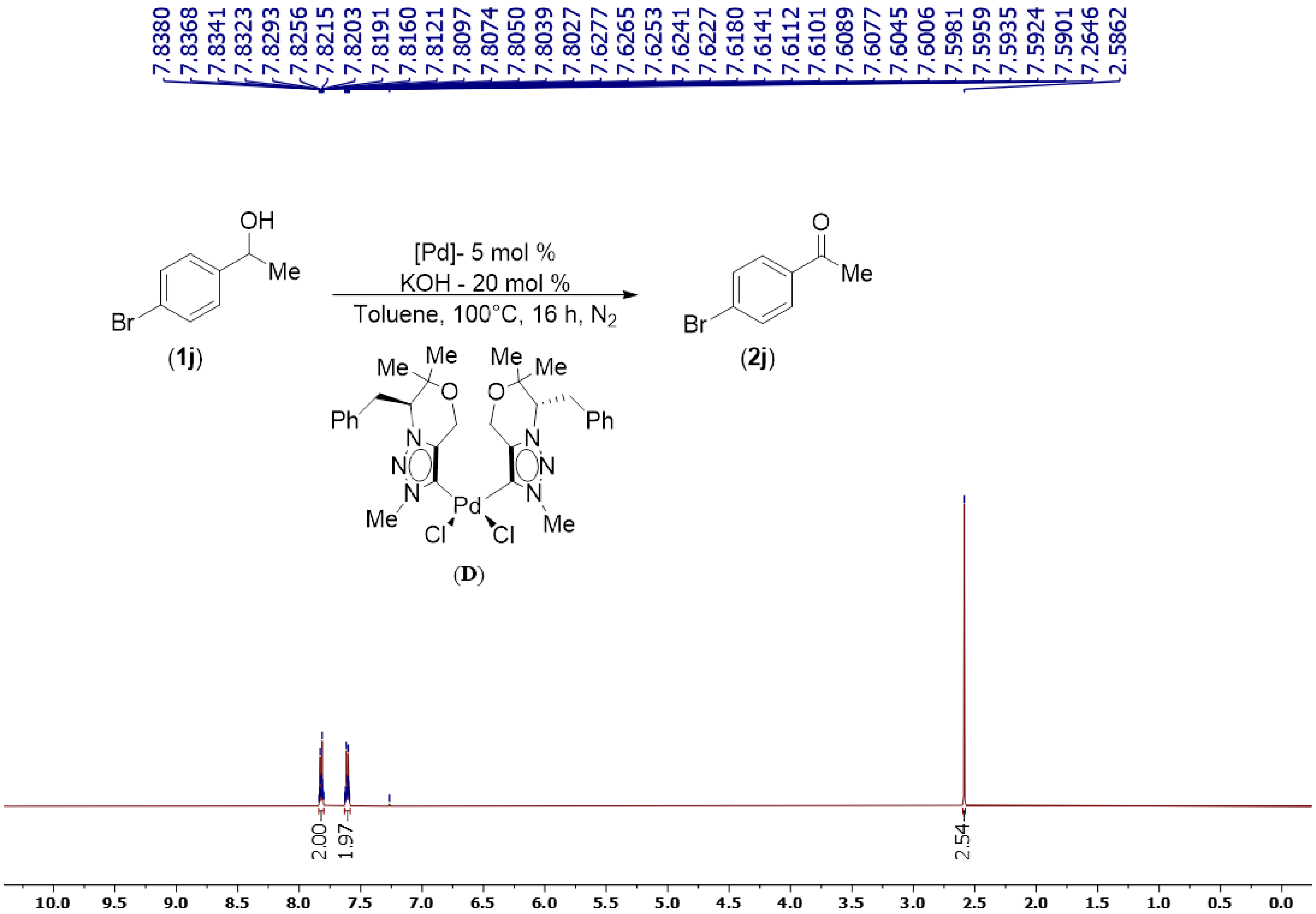

Figure S20. ^{1}H NMR spectrum of **2j** in $CDCl_3$.

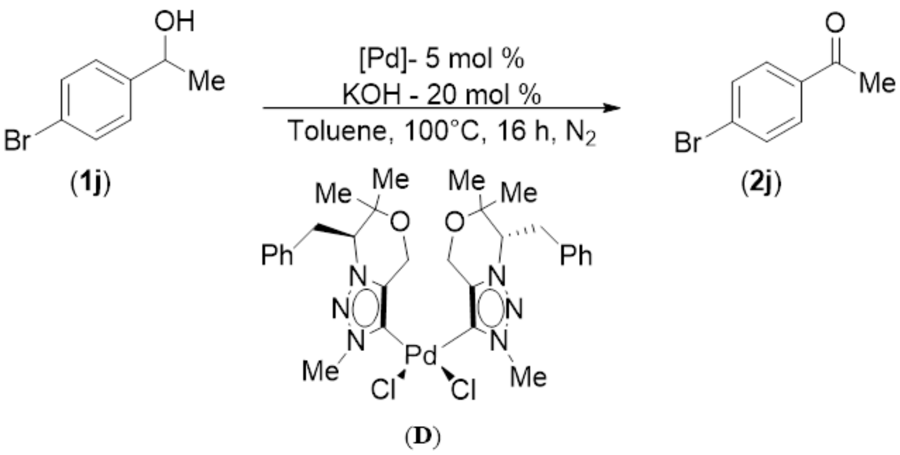

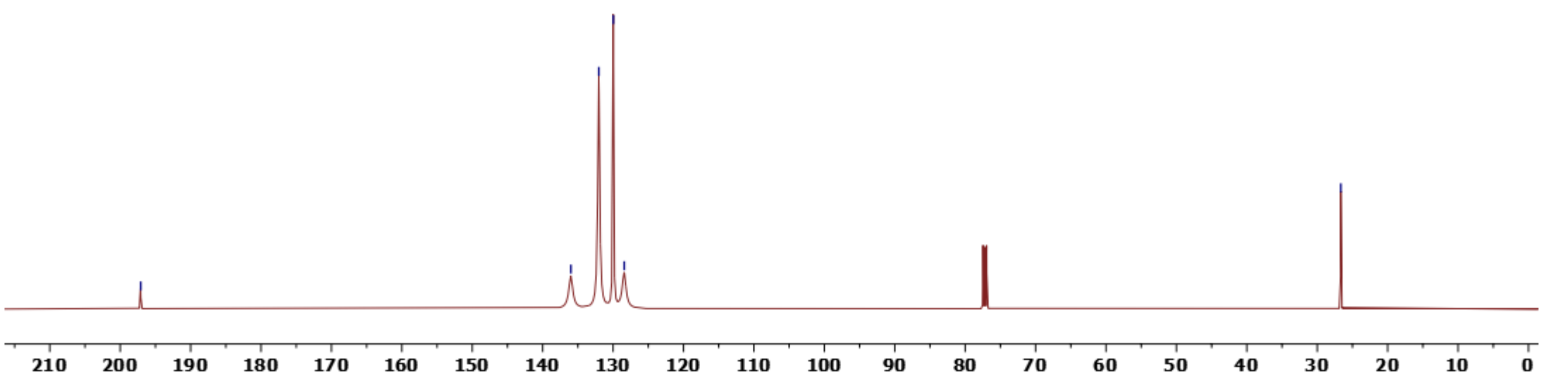

Figure S21. $^{13}C\{^1H\}$ NMR spectrum of **2j** in $CDCl_3$.

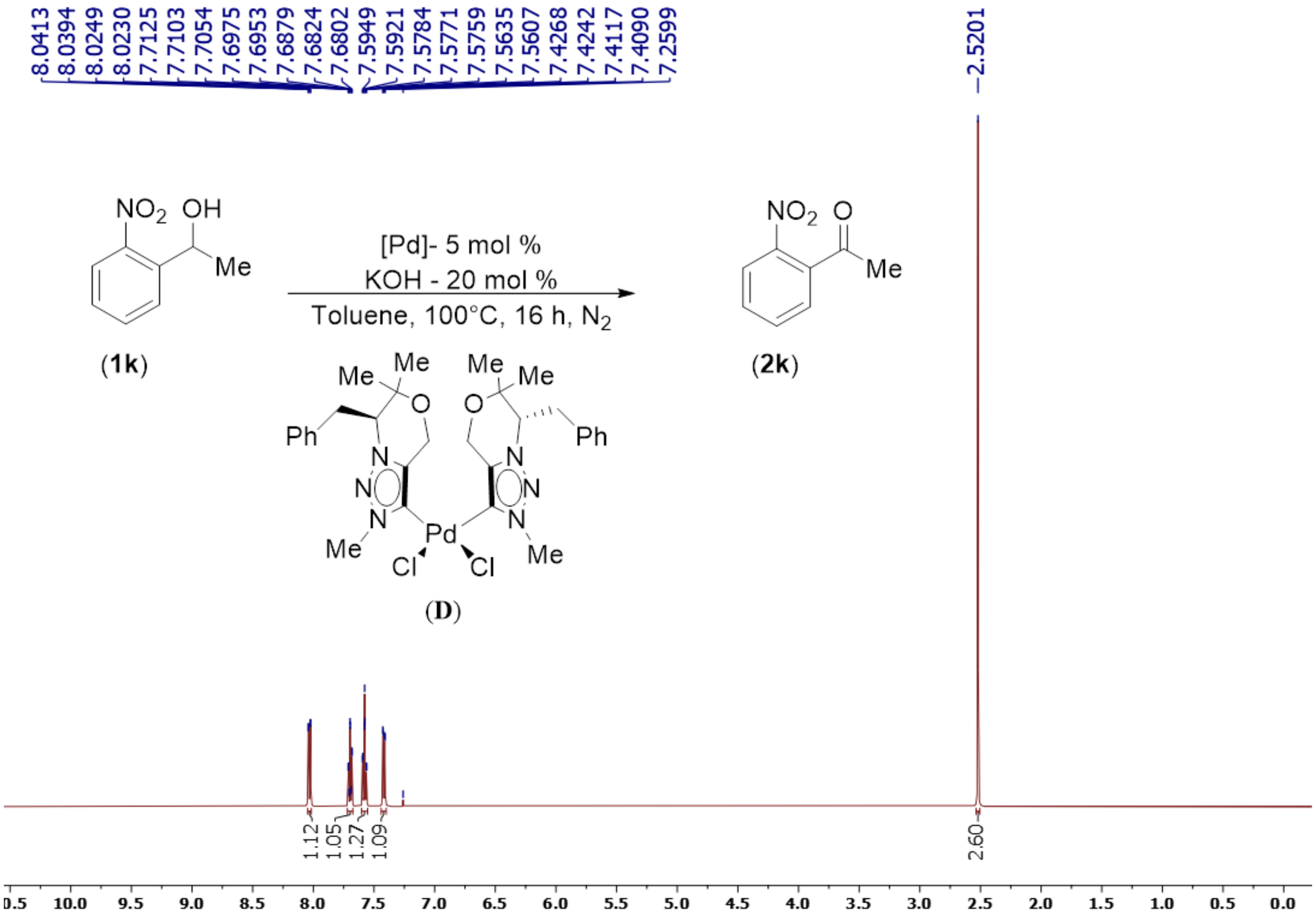

Figure S22. ^{1}H NMR spectrum of **2k** in $CDCl_3$.

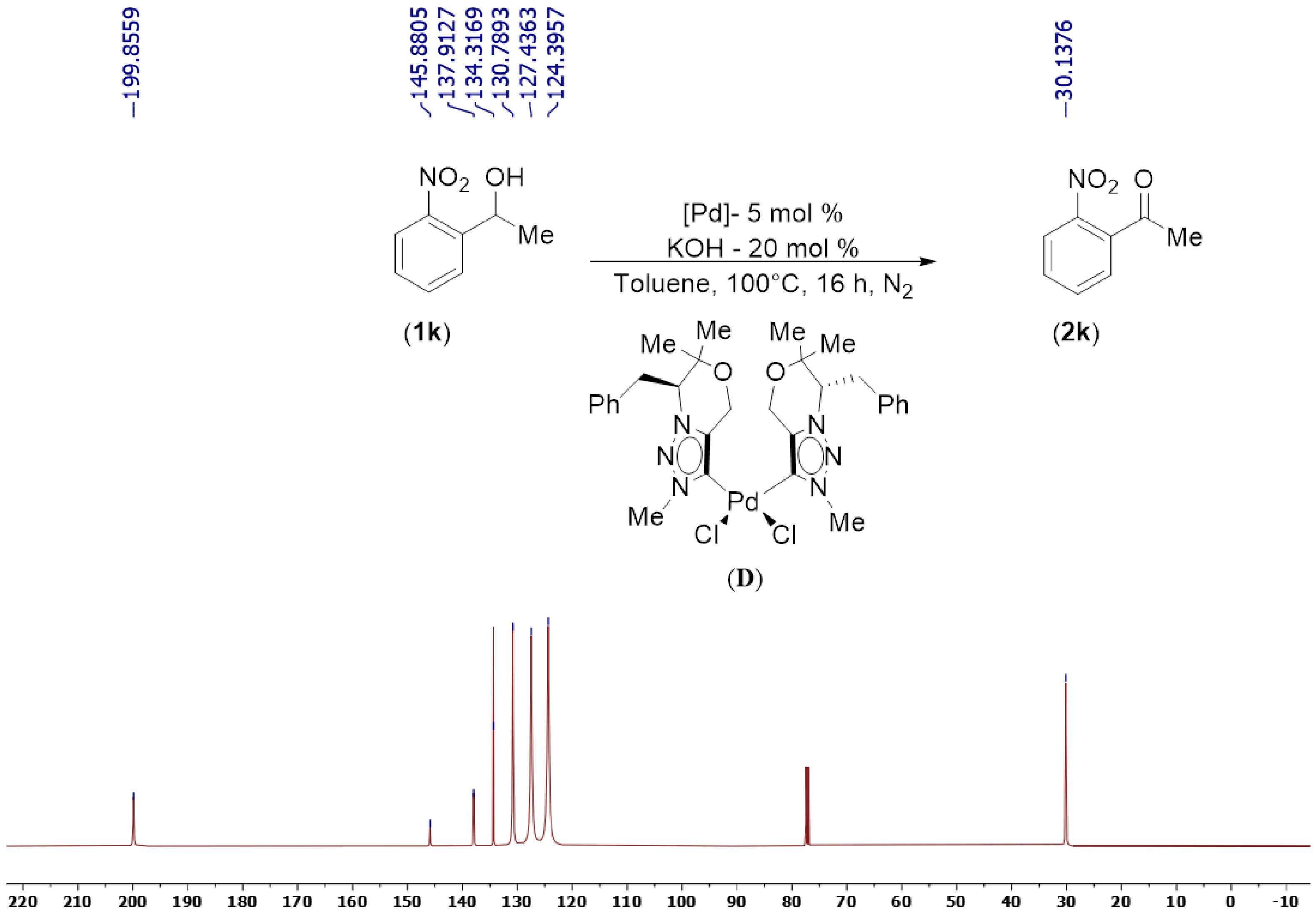

Figure S23. $^{13}C\{^{1}H\}$ NMR spectrum of **2k** in $CDCl_3$.

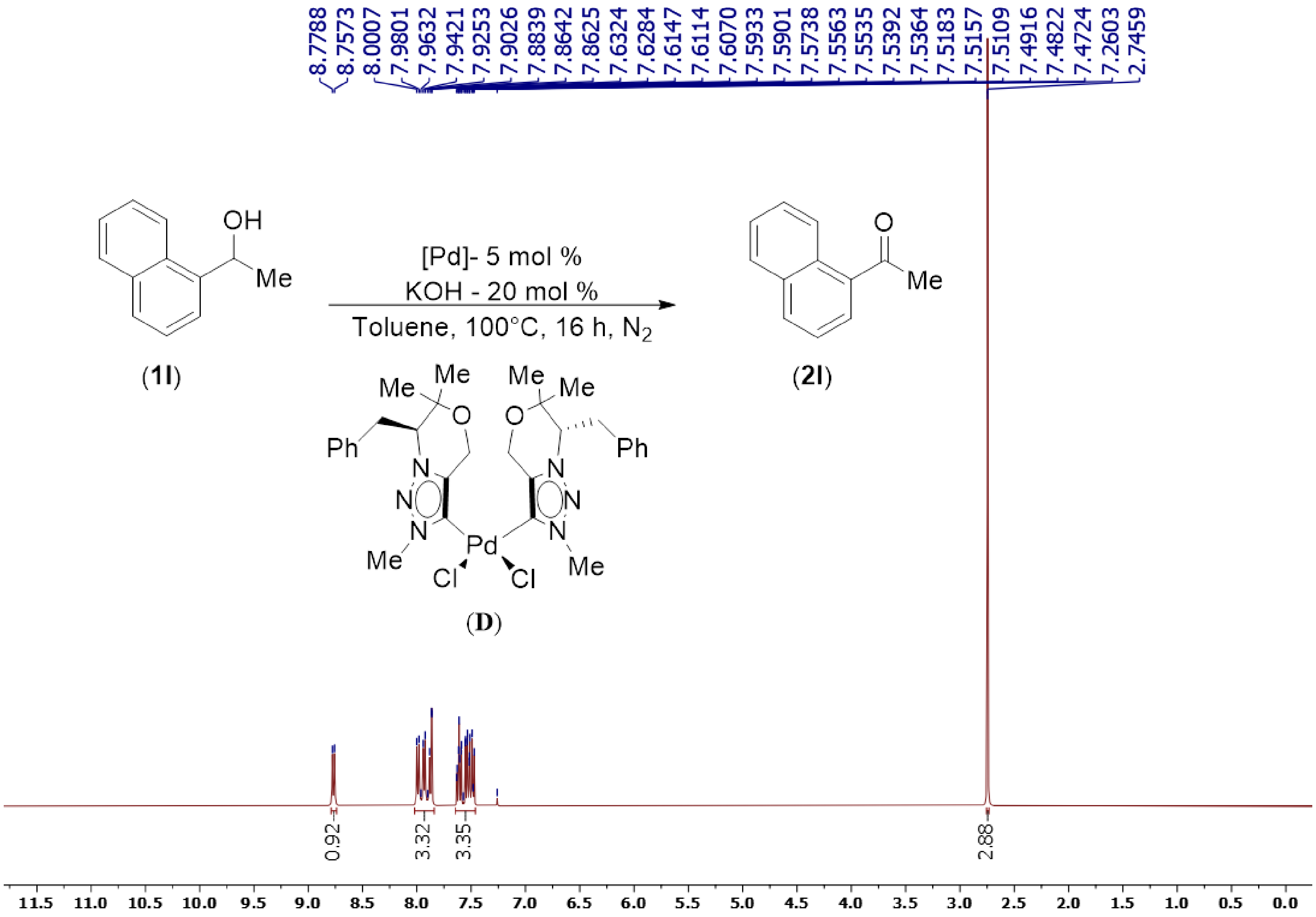

Figure S24. ^{1}H NMR spectrum of **2l** in $CDCl_3$.

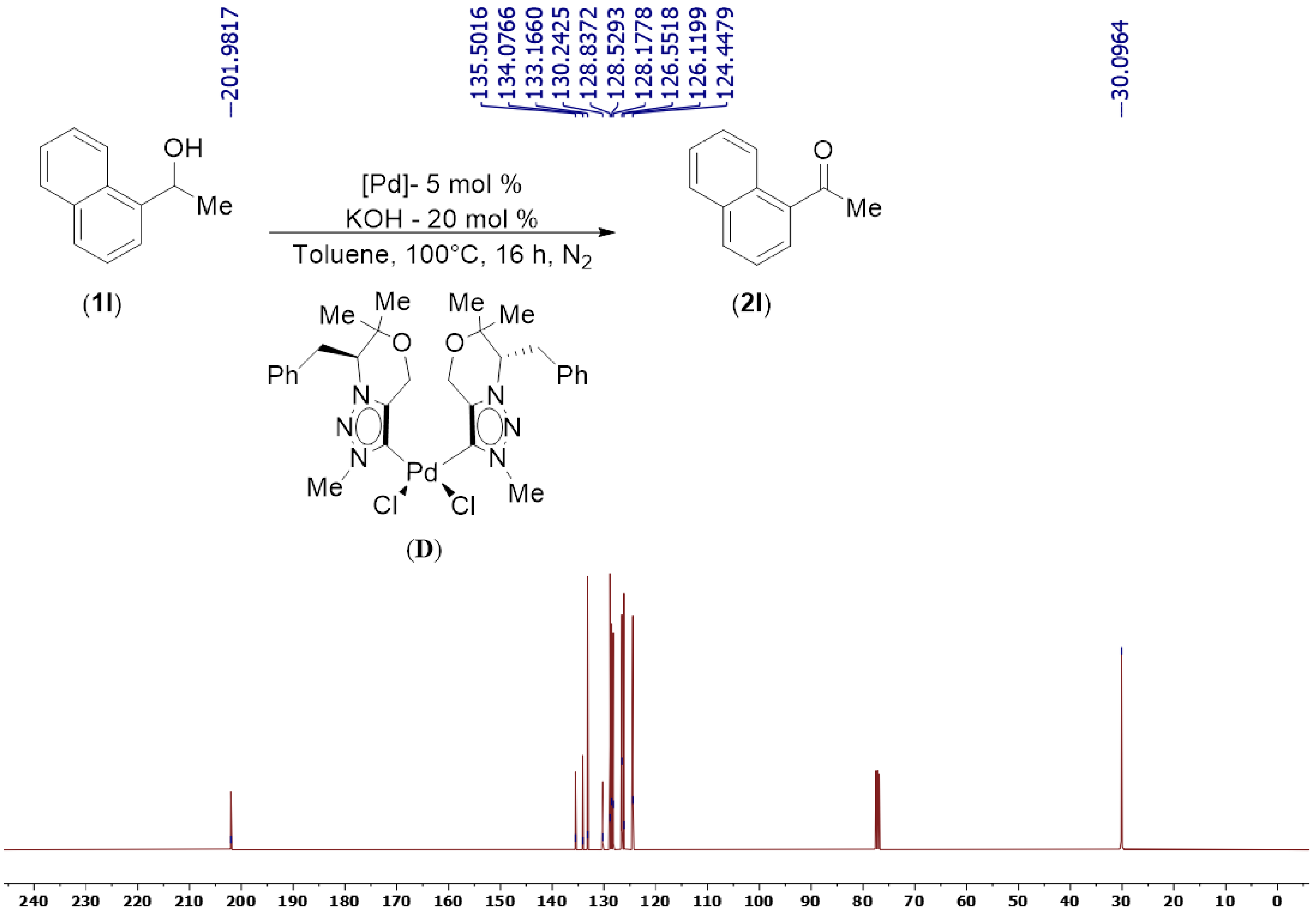

Figure S25. $^{13}C\{^{1}H\}$ NMR spectrum of **2l** in $CDCl_3$.

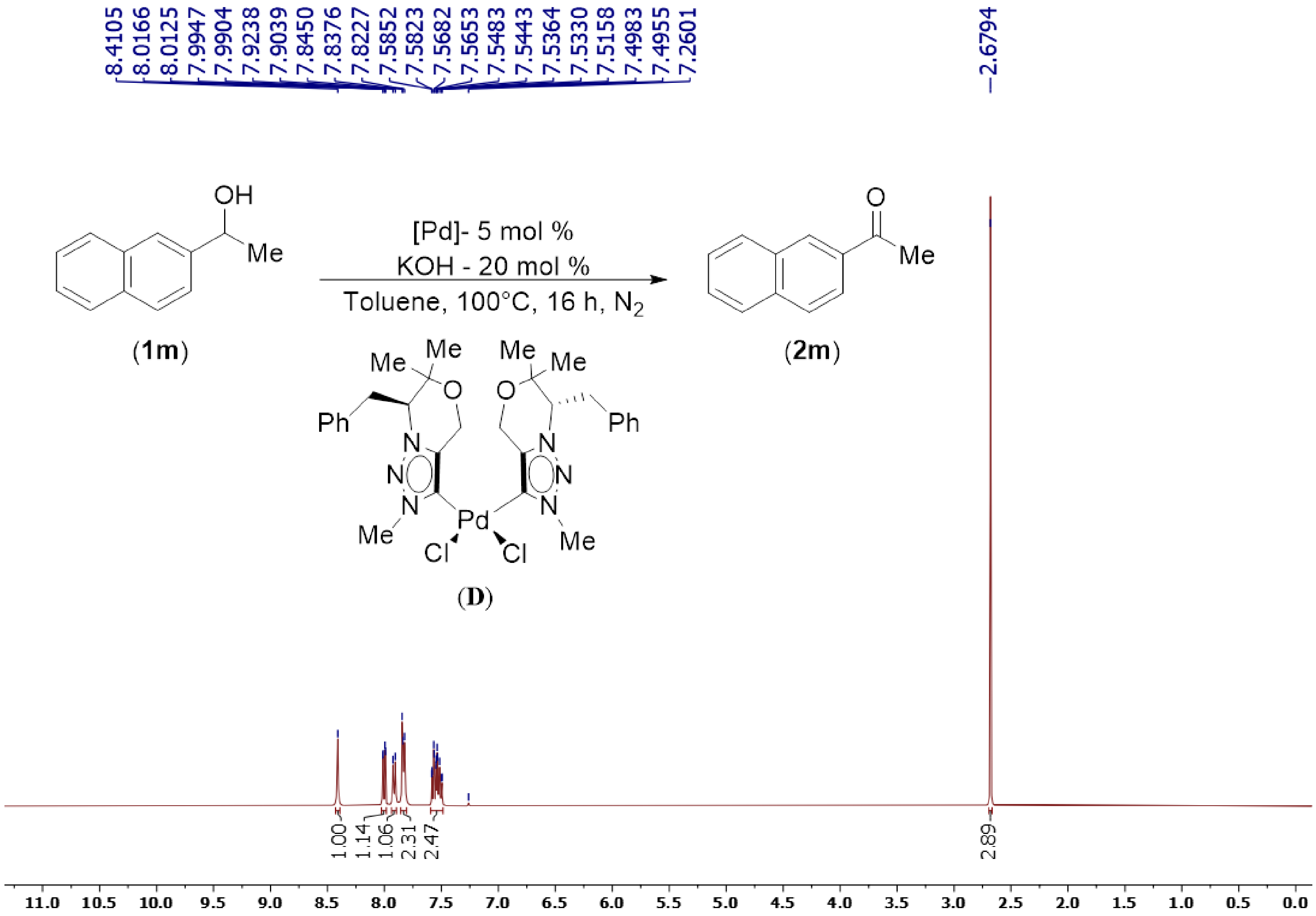

Figure S26. ^{1}H NMR spectrum of **2m** in $CDCl_3$.

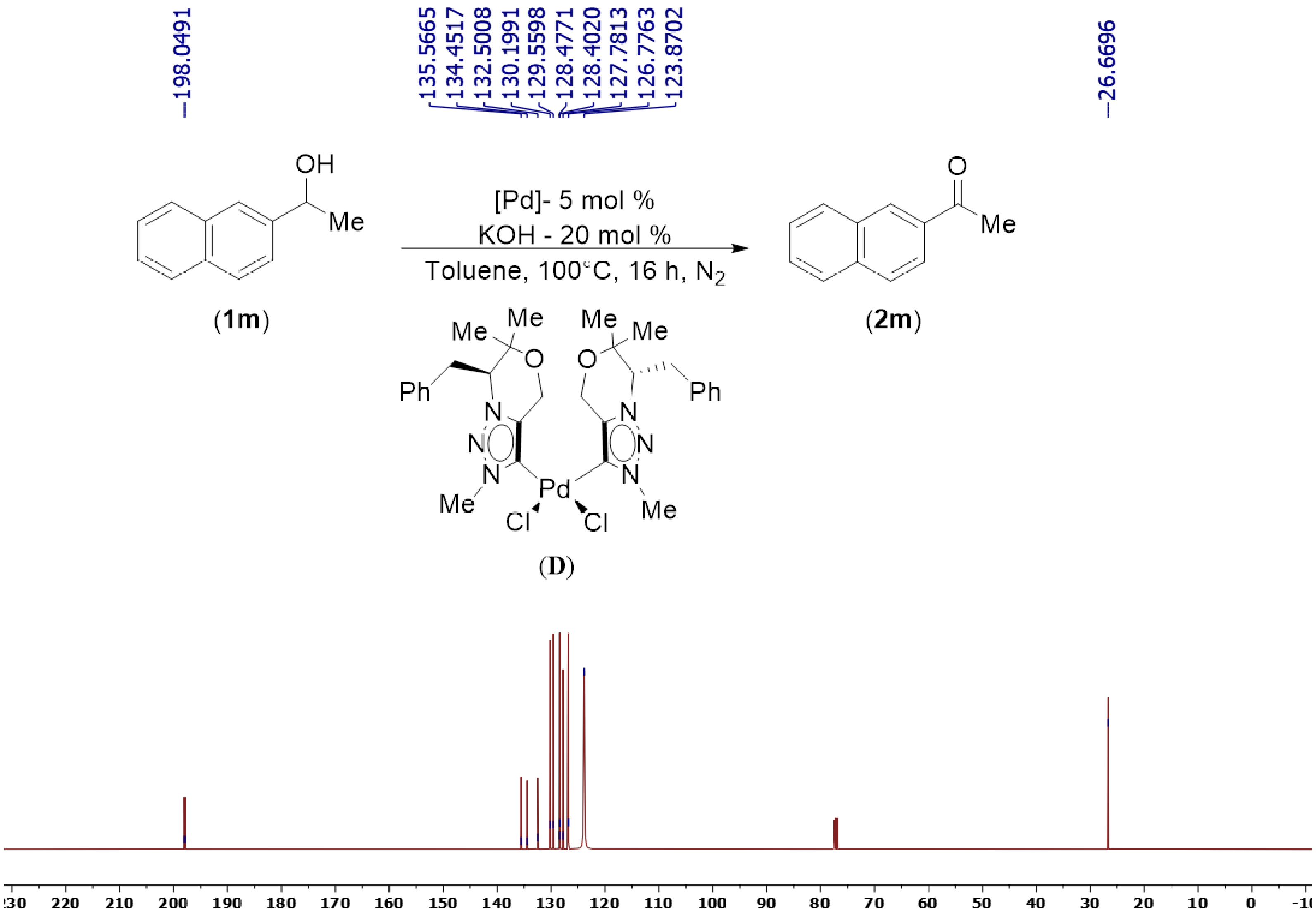

Figure S27. $^{13}C\{^{1}H\}$ NMR spectrum of **2m** in $CDCl_3$.

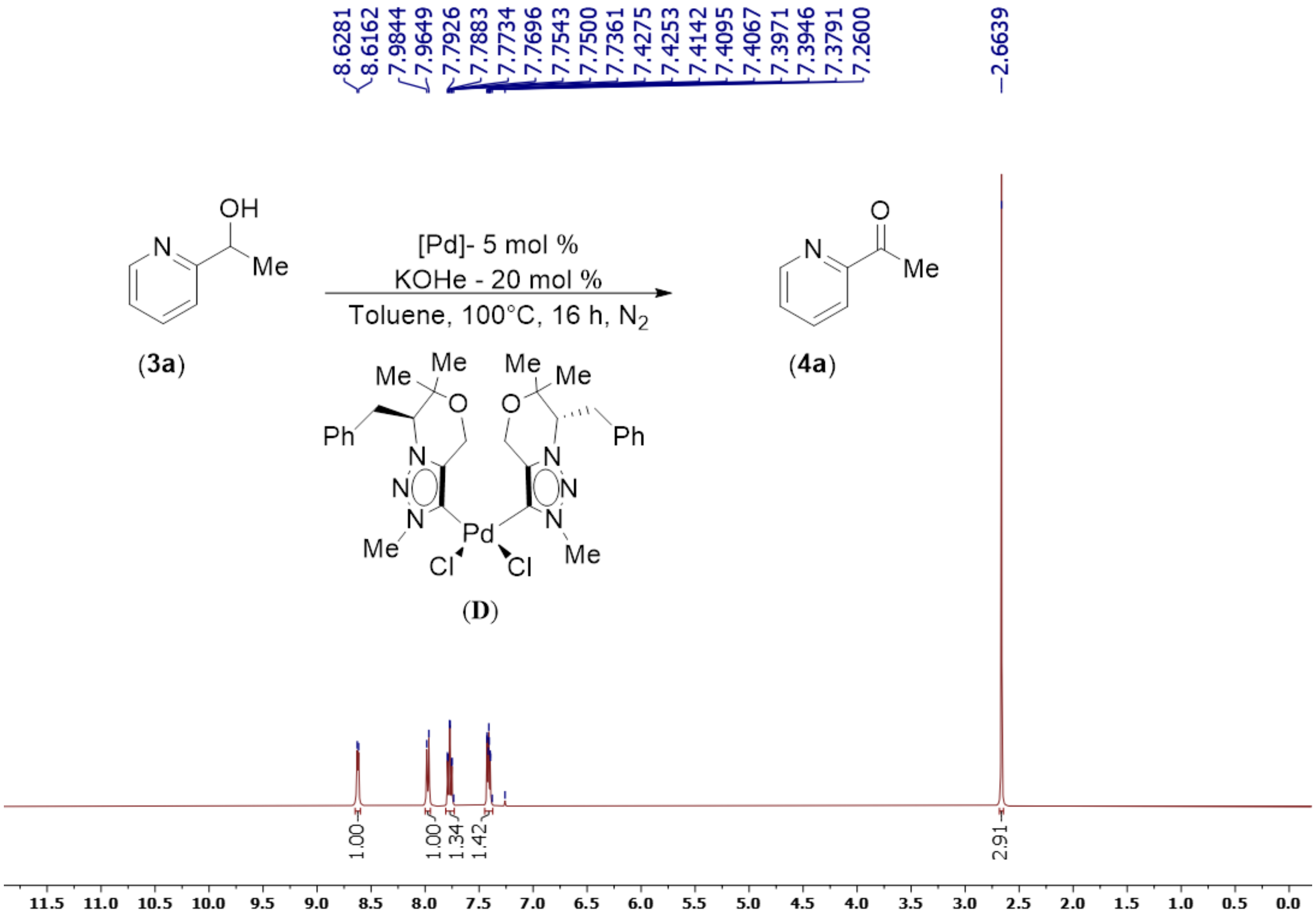

Figure S28. ^{1}H NMR spectrum of **4a** in $CDCl_3$.

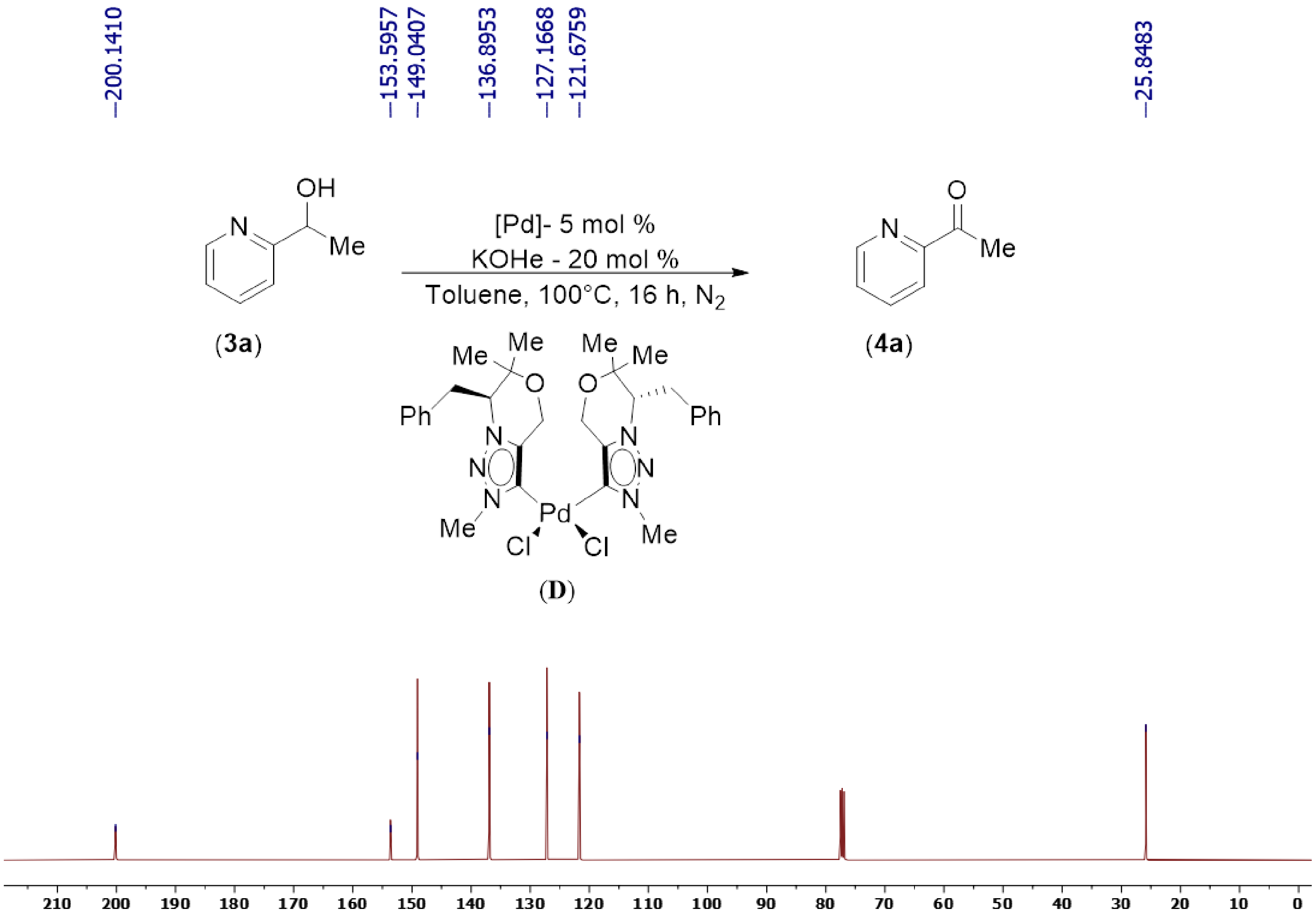

Figure S29. $^{13}C\{^{1}H\}$ NMR spectrum of **4a** in $CDCl_3$.

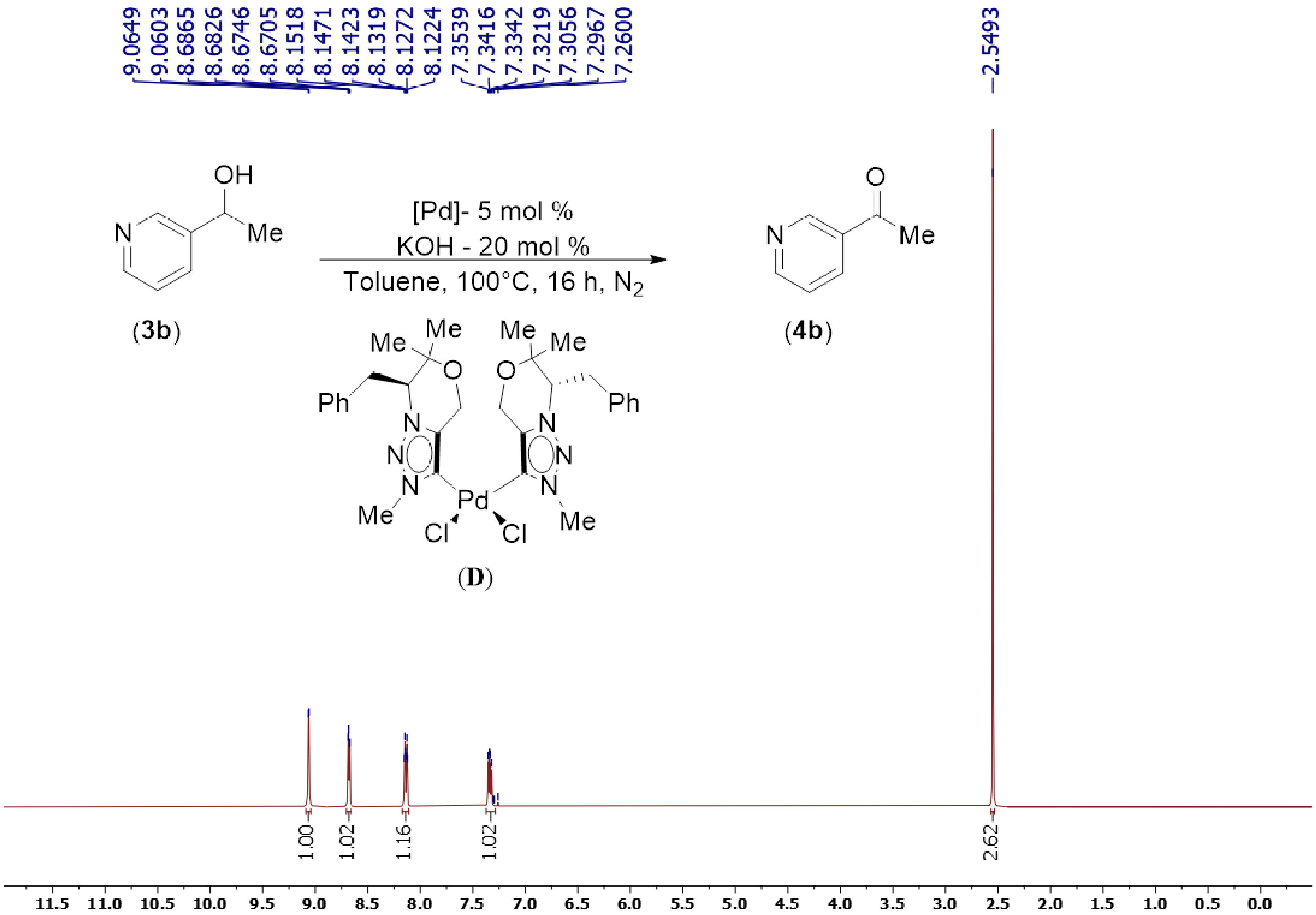

Figure S30. ^{1}H NMR spectrum of **4b** in $CDCl_3$.

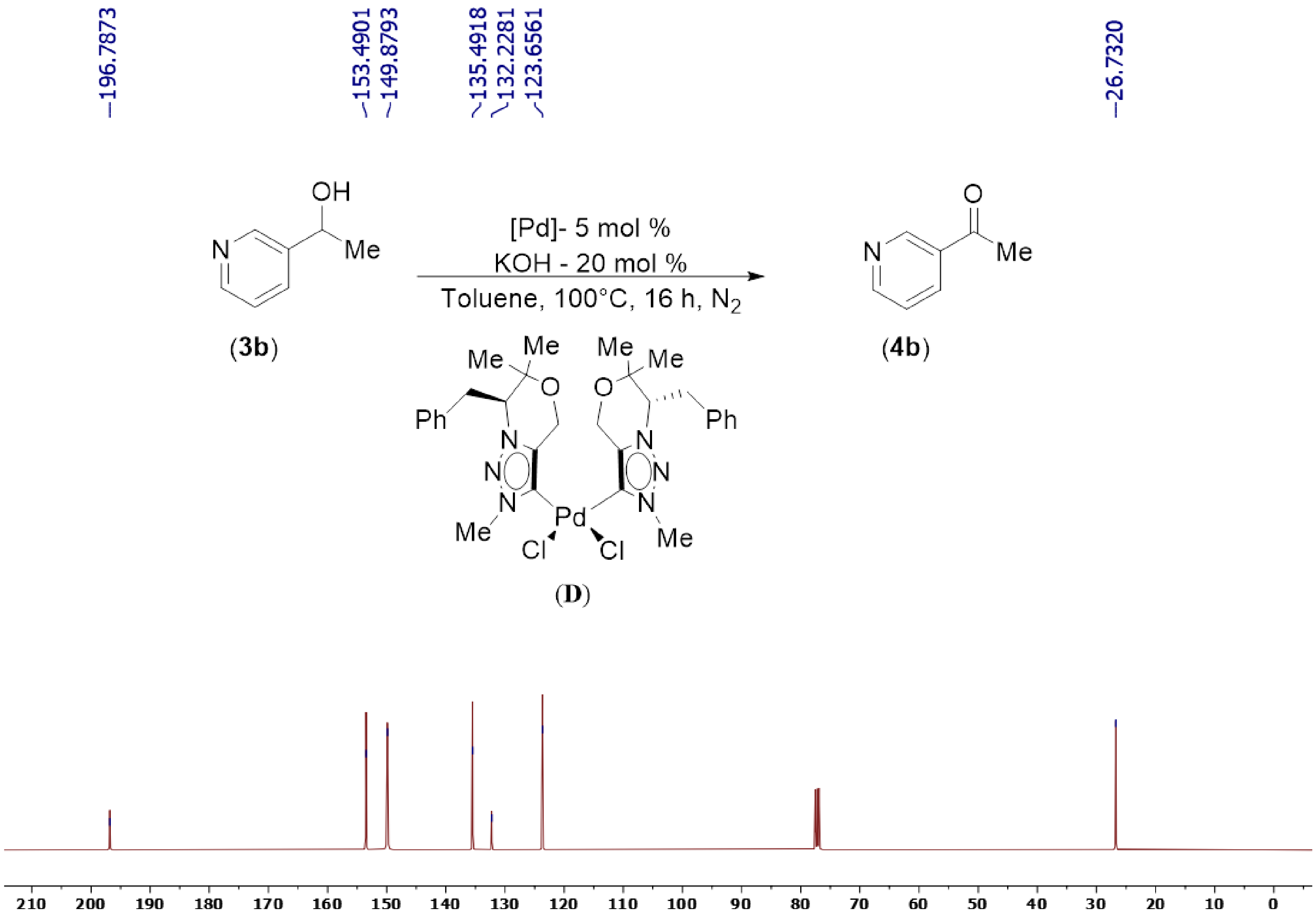

Figure S31. $^{13}C\{^{1}H\}$ NMR spectrum of **4b** in $CDCl_3$.

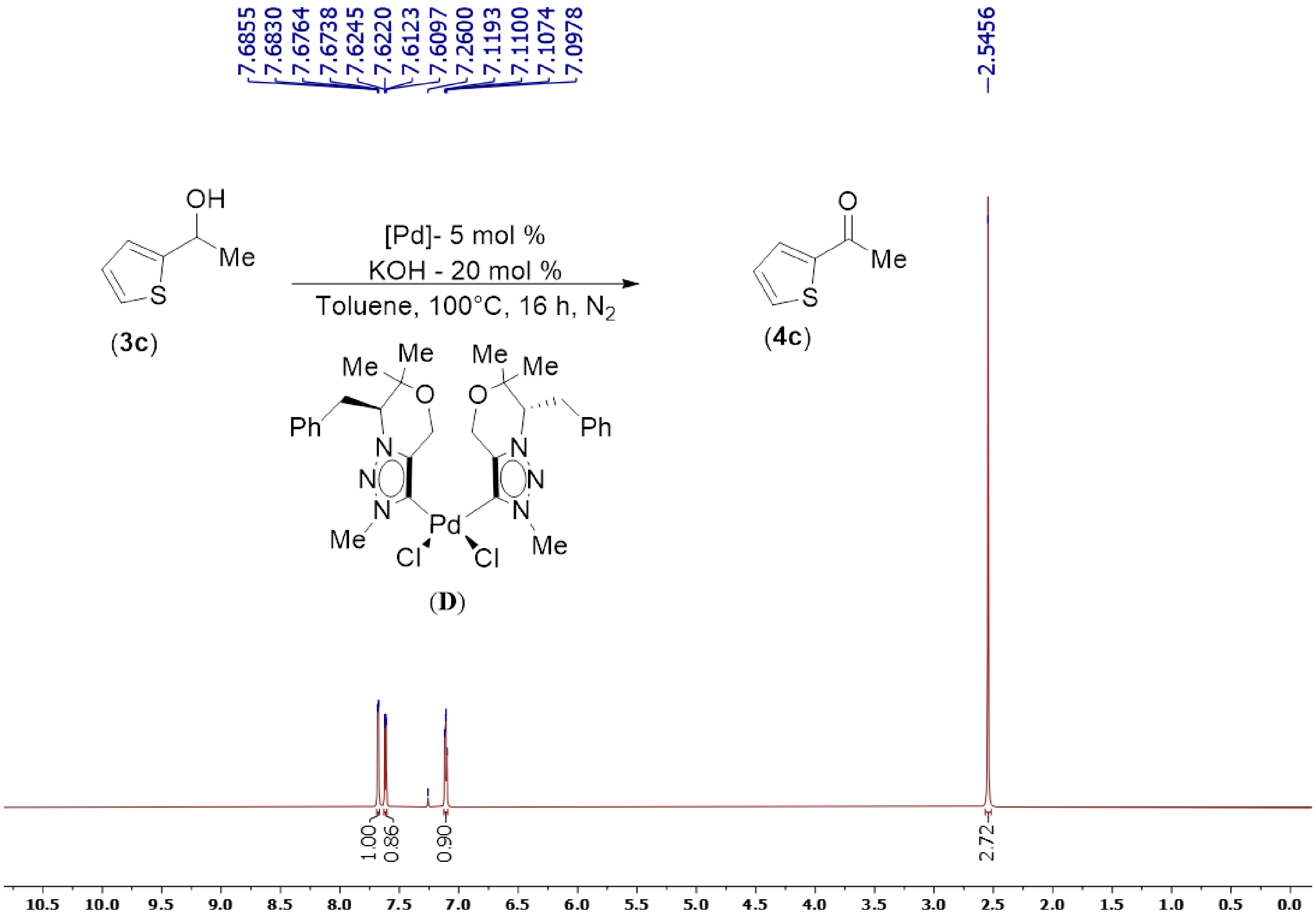

Figure S32. ^{1}H NMR spectrum of **4c** in $CDCl_3$.

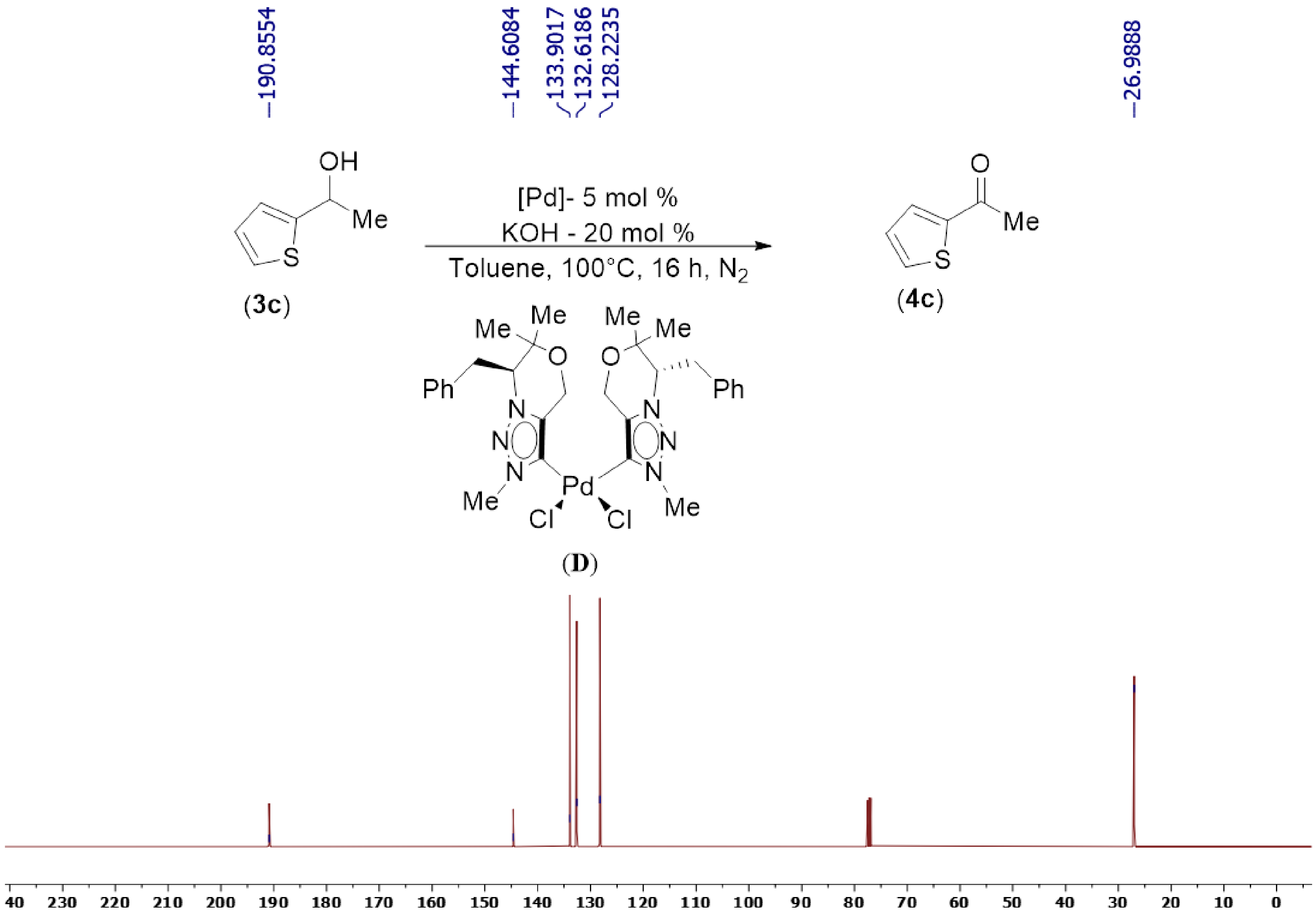

Figure S33. $^{13}C\{^{1}H\}$ NMR spectrum of **4c** in $CDCl_3$.

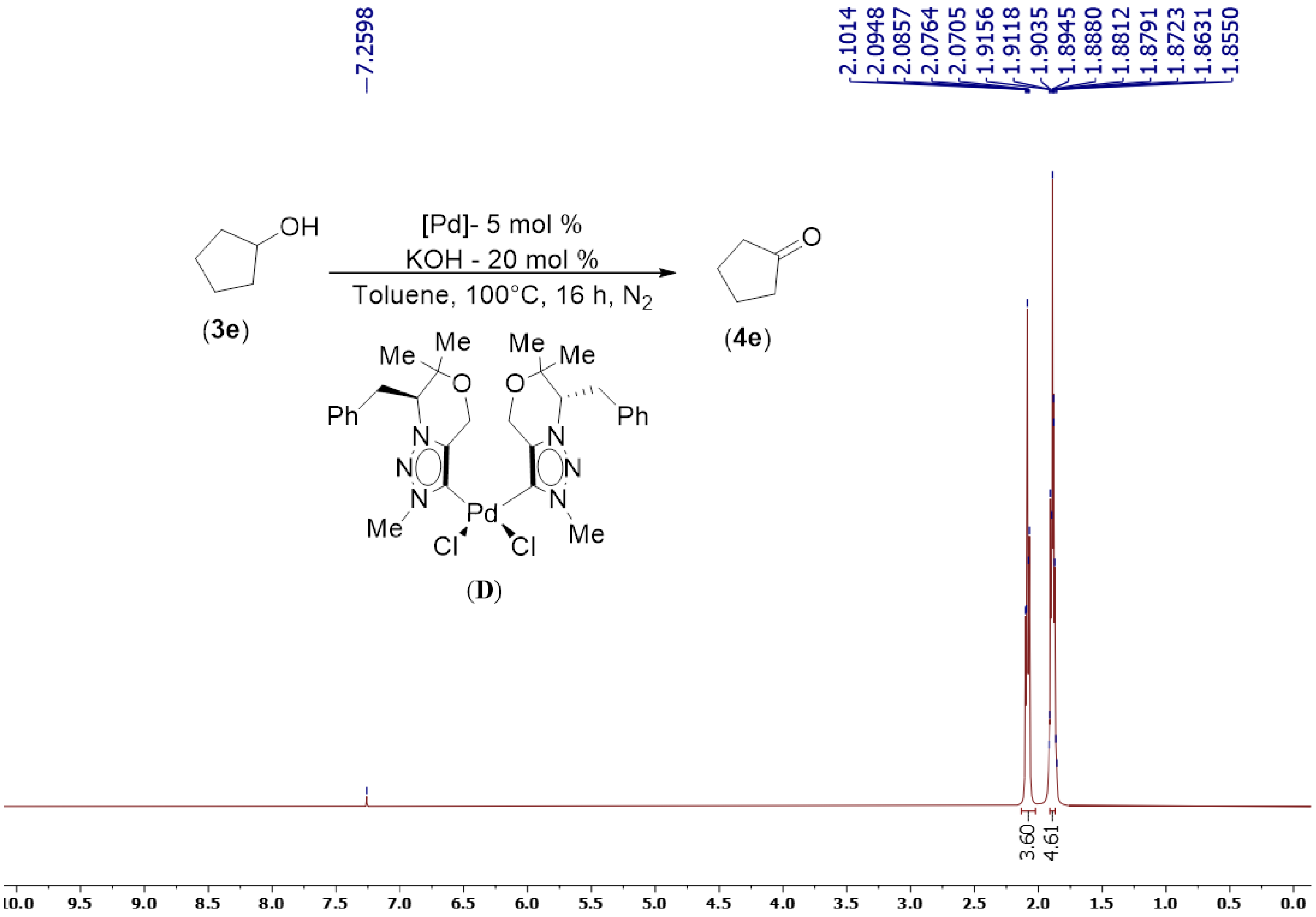

Figure S34. ^{1}H NMR spectrum of **4e** in $CDCl_3$.

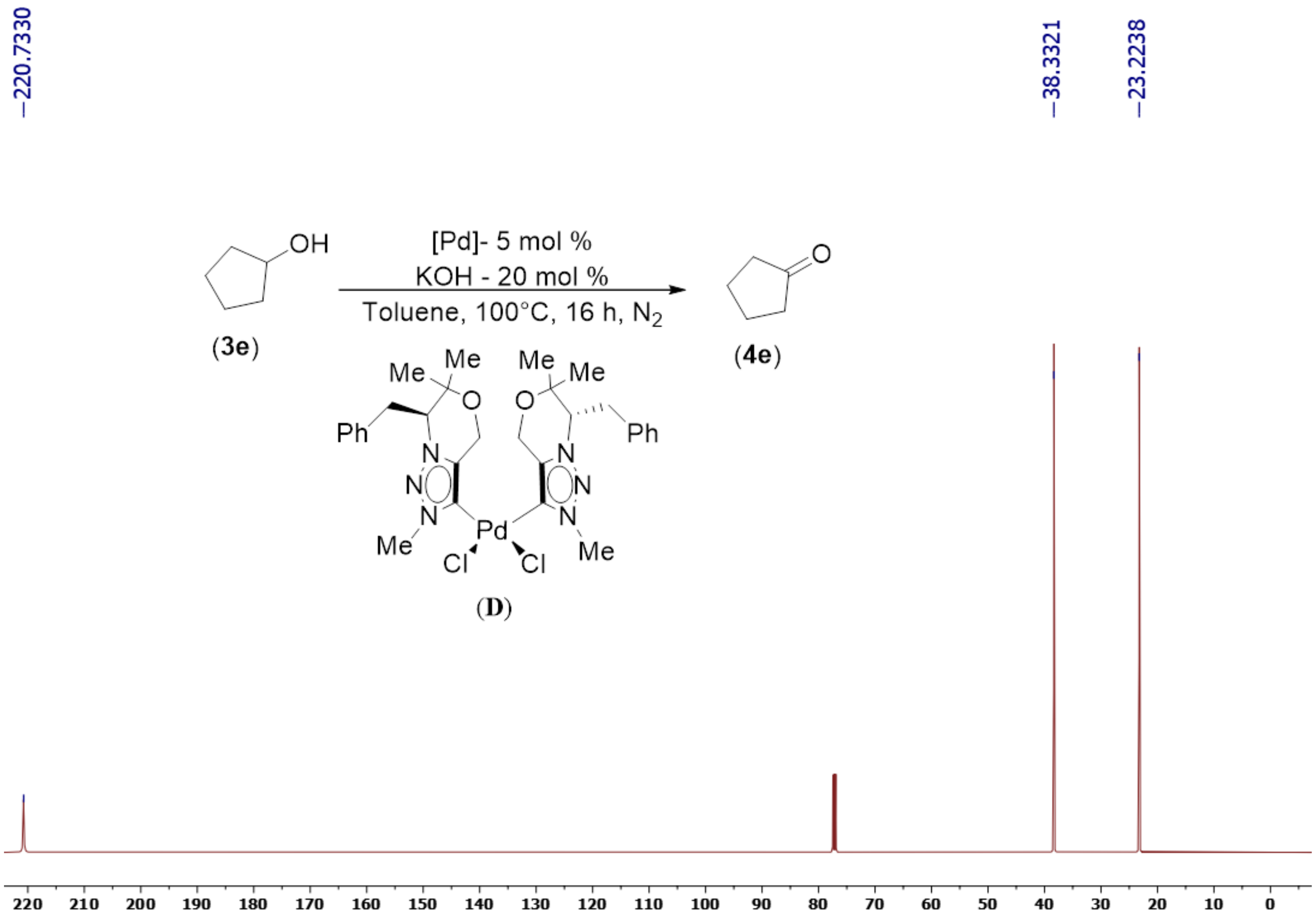

Figure S35. $^{13}C\{^1H\}$ NMR spectrum of **4e** in $CDCl_3$.

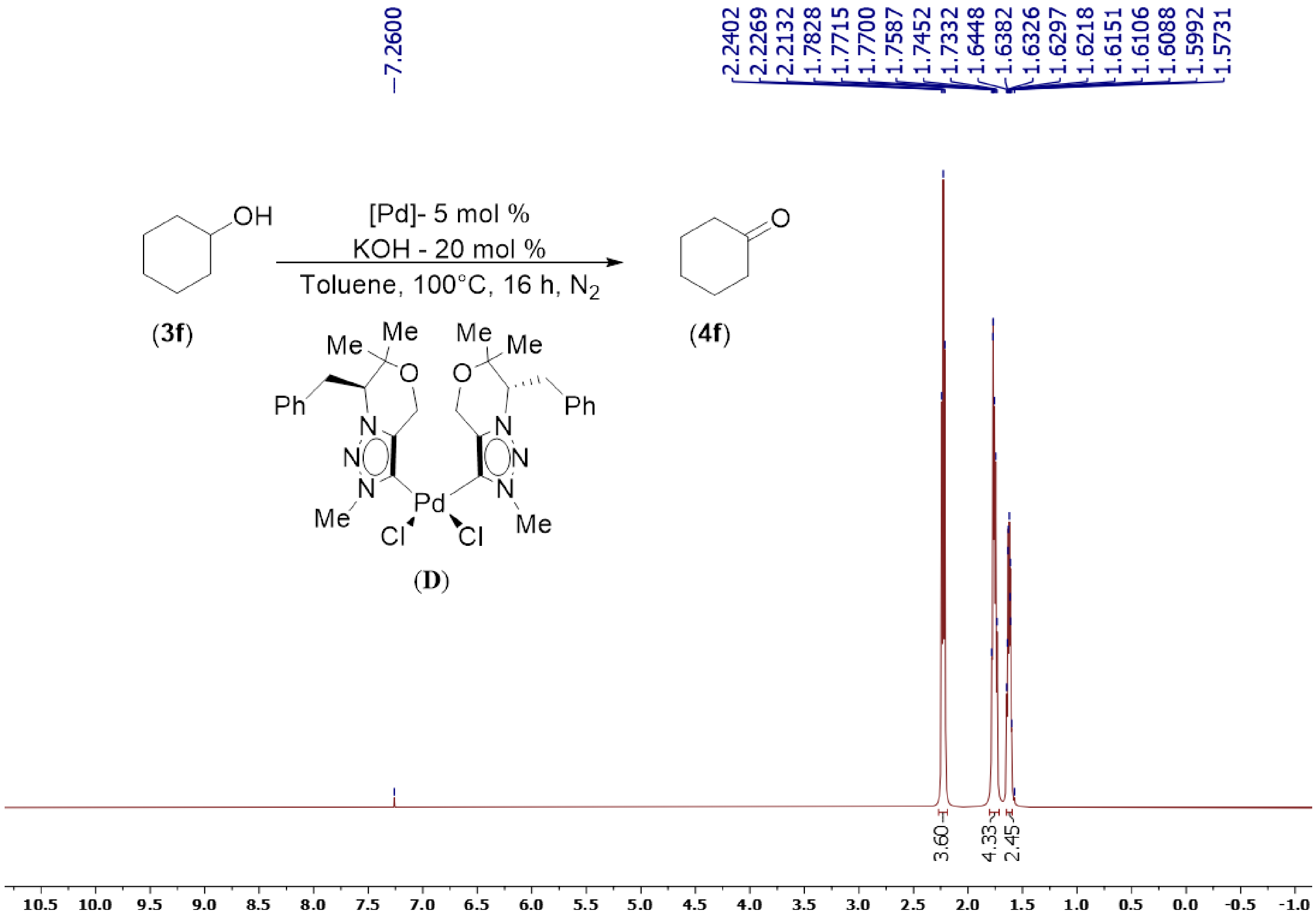

Figure S36. ^{1}H NMR spectrum of **4f** in $CDCl_3$.

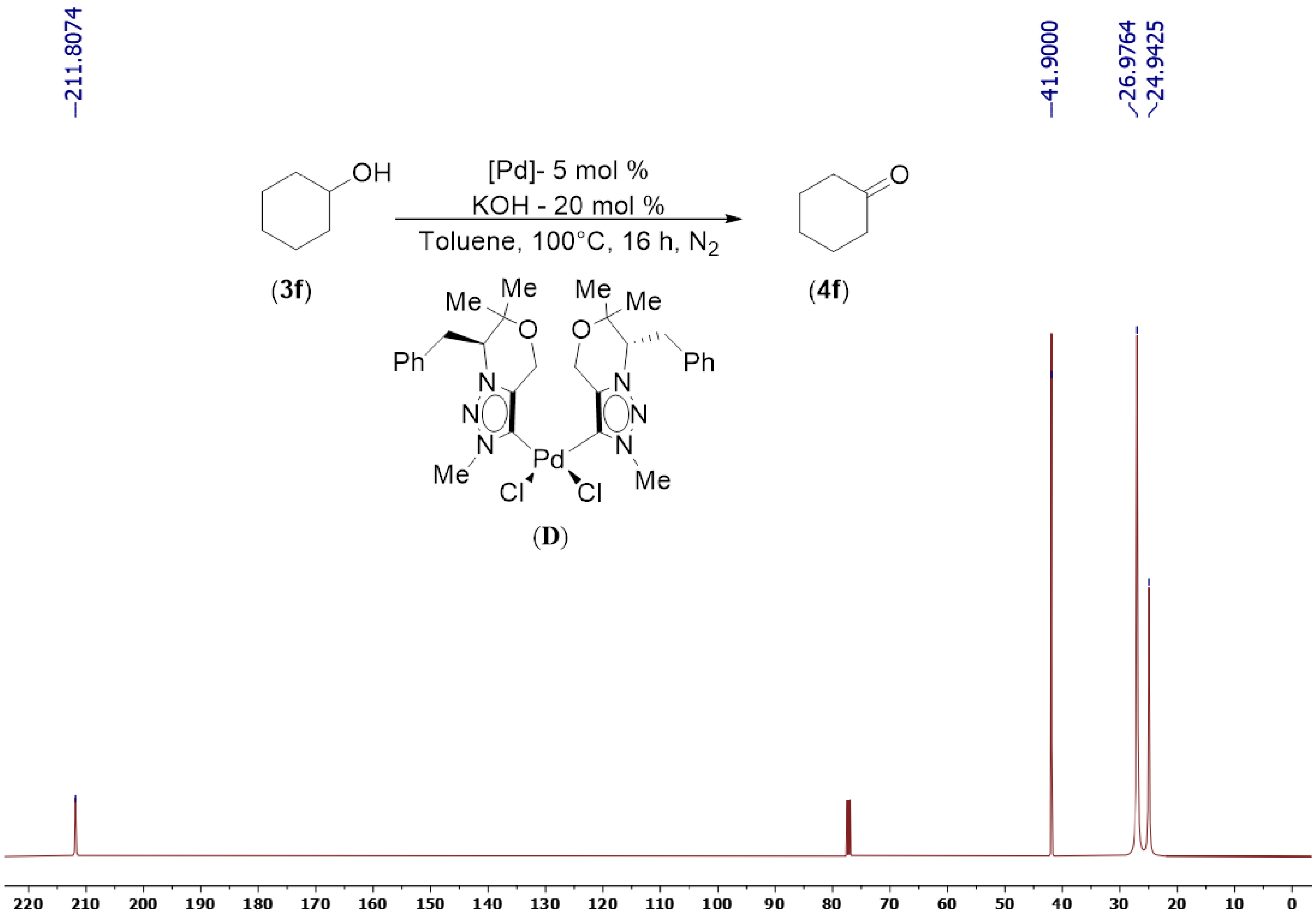

Figure S37. $^{13}C\{^{1}H\}$ NMR spectrum of **4f** in $CDCl_3$.

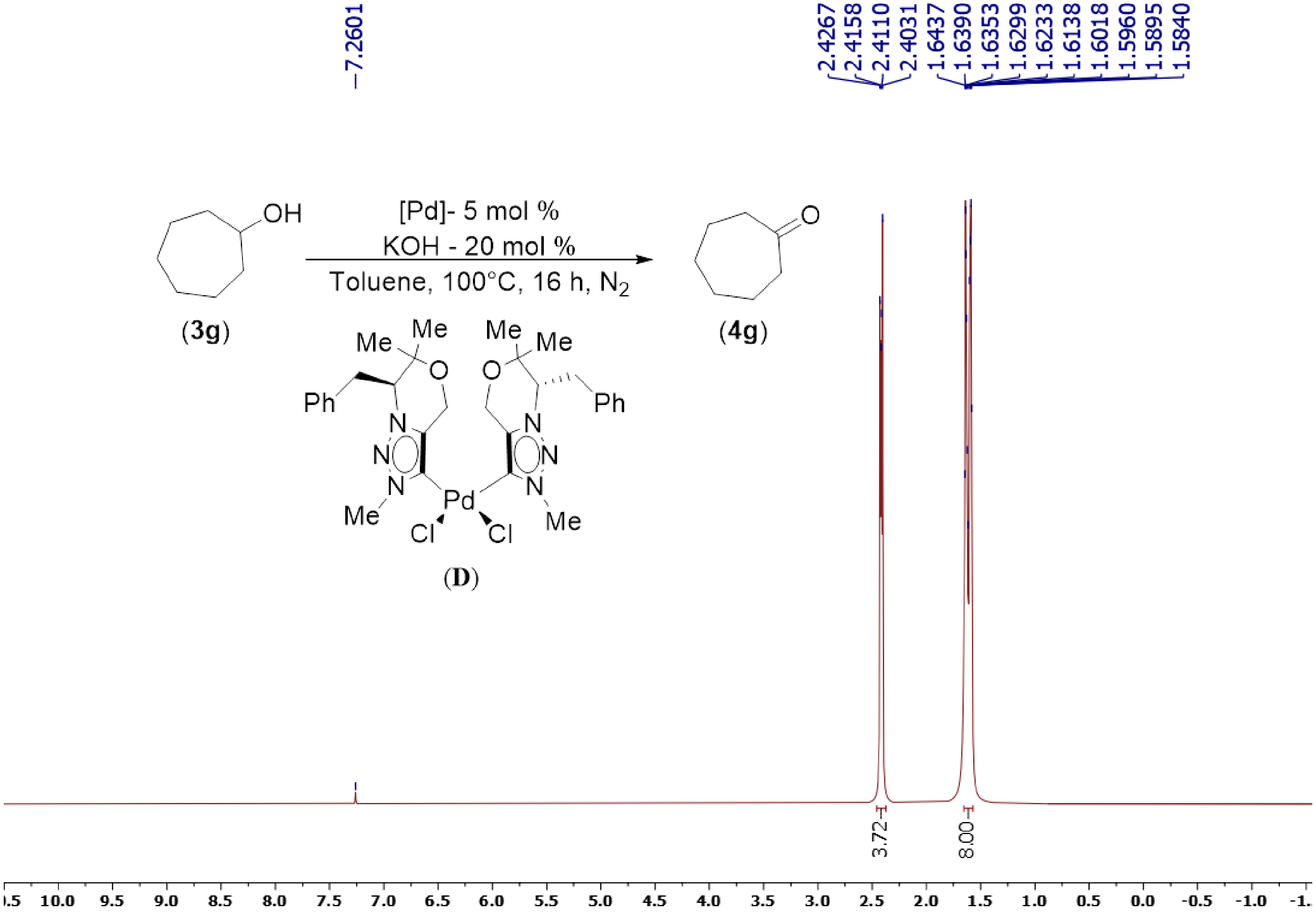

Figure S38. ^{1}H NMR spectrum of **4g** in $CDCl_3$.

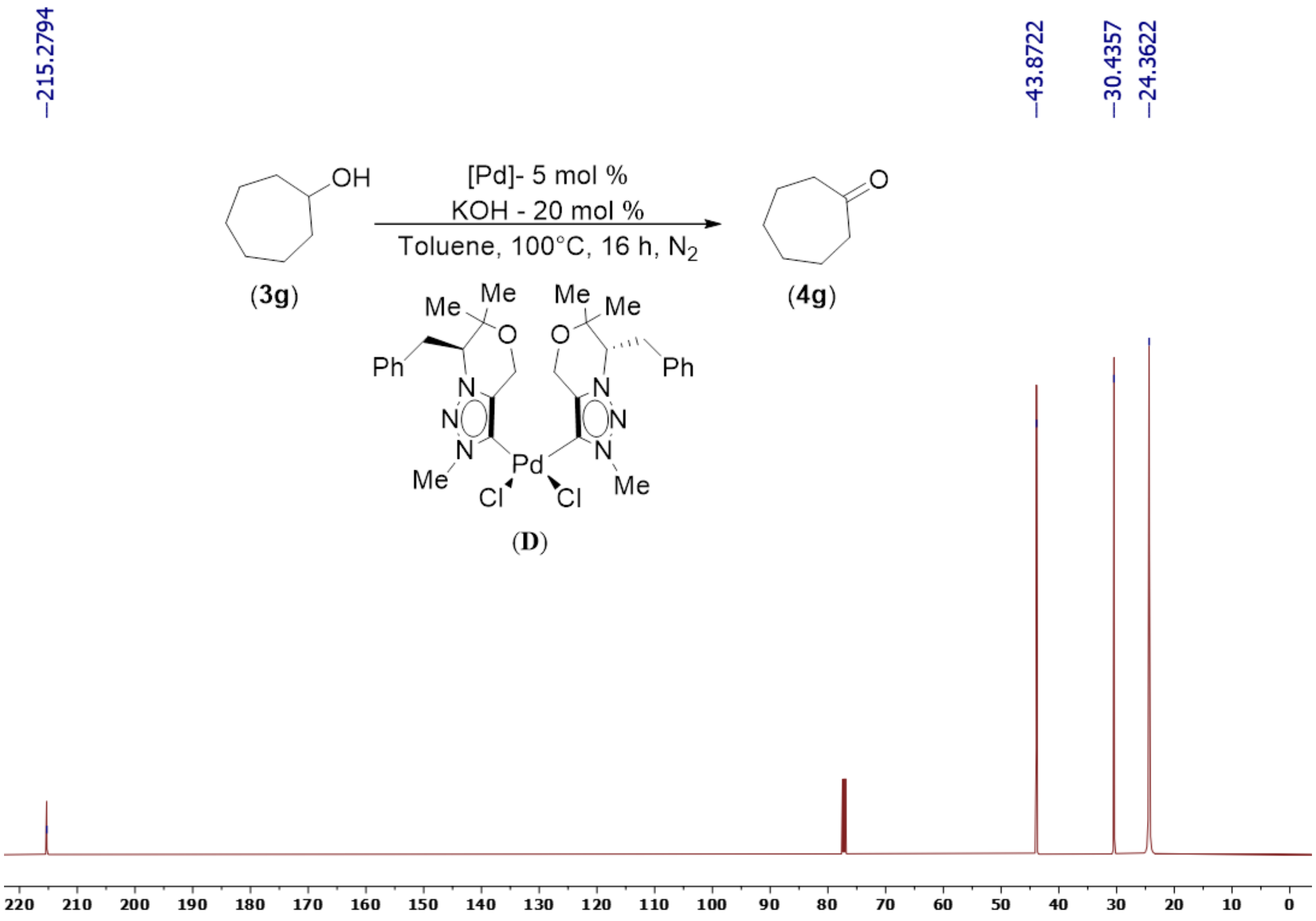

Figure S39. $^{13}C\{^{1}H\}$ NMR spectrum of **4g** in $CDCl_3$.
